# Systematic Review and Meta-Analysis of *Sclerocarya birrea* on Metabolic Disorders: Evidence from Preclinical Studies

**DOI:** 10.3390/metabo14110615

**Published:** 2024-11-12

**Authors:** Desirée Victoria-Montesinos, Pura Ballester, Pablo Barcina-Pérez, Ana María García-Muñoz

**Affiliations:** Faculty of Pharmacy and Nutrition, UCAM Universidad Católica de Murcia, 30107 Murcia, Spain; dvictoria@ucam.edu (D.V.-M.); pbarcina@ucam.edu (P.B.-P.); amgarcia13@ucam.edu (A.M.G.-M.)

**Keywords:** *Sclerocarya birrea*, metabolic disorders, antihyperglycemic, cardiometabolic risk, nutraceuticals, polyphenols

## Abstract

Background/Objectives: Metabolic disorders, including diabetes, obesity, and cardiovascular diseases, are significant global health issues. Nutraceuticals, such as *Sclerocarya birrea* (SB), known for its high polyphenol content, are increasingly explored for managing these conditions. This study aims to evaluate the antihyperglycemic, hypolipidemic, and antihypertensive effects of SB in animal models to understand its potential as a natural intervention for metabolic diseases. Methods: A systematic review and meta-analysis were conducted according to PRISMA guidelines. Searches across databases like PubMed, Web of Science, Embase, and Scopus identified studies using SB in animal models of metabolic disorders. Inclusion criteria were studies with SB intervention, control groups, and quantitative measures of metabolic parameters. The study was registered with INPLASY (INPLASY2024100031). Results: The meta-analysis revealed that SB significantly reduces blood glucose levels in diabetic animal models. Acute administration of SB showed a pooled standardized mean difference (SMD) of −7.13 (95% CI: −11.44 to −2.83) at 1 h and −9.75 (95% CI: −15.92 to −3.59) at 2–4 h post-administration. Chronic administration indicated a non-significant reduction in glucose levels (SMD: −5.69, 95% CI: −16.38 to 5.01). Conclusions: SB appears to have the potential for reducing blood glucose levels and may offer benefits for other cardiometabolic risk factors, including lipid profiles and oxidative stress. However, variability in the results underscores the need for further research, including standardized animal studies and clinical trials, to confirm these effects and clarify the mechanisms by which SB may impact metabolic disorders.

## 1. Introduction

Metabolic disorders, such as diabetes mellitus, obesity, and cardiovascular diseases, continue to rise, presenting significant global health challenges. The International Diabetes Federation estimates that as of 2021, approximately 537 million adults worldwide were living with diabetes, and this number is projected to reach 643 million by 2030 and 783 million by 2045 [1]. Similarly, hypertension remains a pervasive issue, with over 1.28 billion adults affected globally, which substantially increases the risk of cardiovascular events and chronic kidney disease [2]. These conditions are often the result of complex interactions between genetic predispositions and environmental factors, particularly dietary habits [3]. The dysregulation of metabolic pathways is a fundamental contributor to the development and progression of these diseases. As such, there is increasing interest in employing nutrition as an adjuvant in the treatment of these pathologies, specifically using nutraceuticals—bioactive compounds derived from natural sources [4].

Nutraceuticals, a term coined by Stephen L. DeFelice in 1989, refers to food or parts of food that provide medical or health benefits, including the prevention and treatment of disease [5]. These products are designed to be both safe and beneficial, contributing to the management of various health conditions such as colds, sleep disorders, digestive issues, and chronic diseases like diabetes, hypertension, and hypercholesterolemia. Among the different types of nutraceuticals, polyphenols—naturally occurring compounds with strong antioxidant properties—are particularly important. Found abundantly in fruits, vegetables, tea, and whole grains, polyphenols have been associated with numerous health benefits, including improved metabolic health and reduced risk of chronic diseases [6].

*Sclerocarya birrea* (SB), commonly known as marula, is a tree indigenous to Africa, whose fruits, seeds, and leaves have been utilized in traditional medicine to treat a variety of ailments, including metabolic disorders [7]. The potential therapeutic benefits of SB in managing metabolic diseases are largely attributed to its high content of polyphenols, flavonoids, and other bioactive compounds, which exhibit significant antioxidant, anti-inflammatory, and metabolic regulatory properties [8].

Polyphenols, major constituents of SB, are known to positively impact metabolic health by modulating key pathways involved in glucose and lipid metabolism. These compounds have been shown to enhance insulin sensitivity and facilitate glucose uptake by activating AMP-activated protein kinase (AMPK), an essential regulator of cellular energy homeostasis [9]. Additionally, polyphenols can influence the activity of peroxisome proliferator-activated receptor gamma (PPAR-γ), a nuclear receptor that plays a critical role in lipid metabolism and adipocyte differentiation, potentially reducing hyperlipidemia and its associated cardiovascular risks [10].

Beyond AMPK activation, SB may influence glucose metabolism through the inhibition of digestive enzymes, such as alpha-glucosidase and alpha-amylase [11]. Research suggests that phenolic compounds in SB, specifically (−)-epicatechin-3-galloyl ester and gallic acid, are primarily responsible for these inhibitory effects [12,13]. By inhibiting these enzymes, SB might slow the breakdown of carbohydrates in the digestive tract, thereby reducing the absorption of glucose and lowering postprandial blood glucose levels [11]. This approach aligns with current strategies to manage postprandial hyperglycemia, a key factor in the development of type 2 diabetes.

The antioxidant properties of polyphenols in SB are also crucial in mitigating oxidative stress, which is a significant factor in the pathogenesis of insulin resistance and β-cell dysfunction in diabetes. By neutralizing reactive oxygen species and upregulating the activity of endogenous antioxidant defenses, such as superoxide dismutase (SOD) and catalase, polyphenols help protect pancreatic cells from oxidative damage, thereby supporting enhanced insulin regulation and secretion [14].

Currently, there are several preclinical studies on SB focusing on its role in metabolic disorders; however, no meta-analysis has been conducted to quantitatively synthesize its potential beneficial effects. Although research on nutraceuticals, including those targeting metabolic disorders, has shown promising results, the field remains in development, often limiting the number of studies available for meta-analyses [15,16,17].

For this reason, the aim of our study was to conduct a meta-analysis focusing on recent evidence to evaluate the antihyperglycemic, hypolipidemic, and antihypertensive effects of SB. By synthesizing data from various studies, this analysis aims to hypothesize potential mechanisms through which SB may influence metabolic pathways and assess its viability as a natural intervention for metabolic diseases. This research aligns with the broader objective of exploring the interplay between nutrition and metabolic health to improve strategies for the prevention and treatment of metabolic disorders.

## 2. Materials and Methods

This systematic review and meta-analysis were conducted following the guidelines set forth by the Preferred Reporting Items for Systematic Reviews and Meta-Analysis (PRISMA) statement [18]. This systematic review and meta-analysis protocol has been registered in the International Platform of Registered Systematic Review and Meta-analysis Protocols (INPLASY) with the registration number INPLASY2024100031.

### 2.1. Search Strategy

A thorough literature search was performed across multiple electronic databases, including PubMed, Embase, Web of Science, and Scopus, to identify studies published up to [specific date]. The search terms included both MeSH and non-MeSH keywords relevant to the topic, such as “*Sclerocarya birrea*”, “marula oil”, “marula fruit”, “marula seed”, “marula tree”, “*S. birrea*”, and terms related to various metabolic conditions, including “metabolic syndrome”, “syndrome X”, “insulin resistance”, “prediabetes”, “type 2 diabetes”, “hypertension”, “blood pressure”, “vasodilation”, “obesity”, “overweight”, “inflammation”, “oxidative stress”, “dyslipidemia”, “cholesterol”, “triglycerides”, “glucose metabolism”, “insulin sensitivity”, “cardiovascular health”, and “glycaemia”. No restrictions were applied regarding the type of publication, language, study design, sample size, or publication date. Additionally, reference lists of selected studies and relevant review articles were screened for additional citations. The bibliographic records were managed, and duplicates were removed using Zotero software (version 7.0.8 for Windows 10; Corporation for Digital Scholarship).

### 2.2. Elegibility Criteria

Studies were included if they met the following criteria: (1) involved animal models of metabolic conditions such as diabetes or insulin resistance, (2) included administration of SB or its derivatives, (3) used a control group for comparison, and (4) provided quantitative or qualitative measurements of fasting glucose, glucose levels following glucose consumption, or other cardiovascular health-related parameters. Studies that were in vitro, clinical trials, review articles, case reports, letters, abstracts from conferences, or involved the effects of SB combined with other plants were excluded.

### 2.3. Data Extraction

Following the identification of potential studies, we utilized Mendeley (Windows 10 version; Elsevier, Amsterdam, The Netherlands) to remove duplicate entries. Two independent reviewers (D.V.-M. and A.M.G.-M.) initially screened the titles and abstracts of all retrieved articles to identify relevant studies for full-text review. In cases of disagreement, a third researcher (P.B.) performed a final assessment. Selected articles were then evaluated in full to confirm eligibility based on the established criteria. Data extraction was performed using a standardized form, capturing information such as the author’s name, country, year of publication, study design, animal type and characteristics, method of diabetes induction, dosage, route of administration, and duration of intervention. Quantitative data extracted included the mean and standard deviation (SD) of fasting glucose levels and changes in glucose levels after glucose consumption at specified time intervals. When data were available only in graphical format, values were extracted using the AutoMeris WebPlotDigitizer software (version 5.2 for Windows 10) [19]. For studies with multiple treatment doses, each dose was analyzed independently against the control group.

### 2.4. Quality Assessment

The quality assessment of the included studies was conducted using SYRCLE’s risk of bias tool [20], specifically designed for animal intervention studies. This tool evaluates a comprehensive set of 14 domains to provide an in-depth assessment of the methodological quality of each study. The domains include sequence generation, which examines whether a random sequence method was used to allocate animals to different groups, and baseline characteristics, which assesses if the groups were similar at the start of the experiment or if confounding factors were adjusted. Allocation concealment considers whether the allocation process was adequately concealed to prevent selection bias, while random housing evaluates if animals were housed randomly to avoid performance bias. Blinding of caregivers and investigators is assessed to determine if those administering the interventions were unaware of the group assignments, thereby reducing performance bias, and random outcome assessment checks if the selection of animals for outcome assessment was random to prevent detection bias. The tool also examines the blinding of outcome assessors to ensure that those evaluating the outcomes are blinded to the intervention groups, reducing detection bias. Incomplete data outcomes are evaluated to see how missing data were handled and if all animals were accounted for in the results, while selective reporting checks for the presence of reporting bias by ensuring that all pre-specified outcomes are reported. Additional domains under “other sources of bias” include assessing the study’s freedom from contamination, inappropriate influence of funders, and unit of analysis errors. It also considers design-specific risks of bias and whether new animals were added to replace those that dropped out, which could introduce bias. Each domain is rated as low, high, or unclear risk, providing a comprehensive overview of the potential biases and methodological rigor of each study. To create a graphical representation of the risk of bias assessment, Microsoft Excel was used.

### 2.5. Statistical Analysis

Meta-analyses were performed for outcomes with data available from at least three independent studies. The standardized mean difference (SMD) and 95% confidence intervals (95% CI) were calculated for each intervention-control comparison. A random-effects model was used to account for anticipated heterogeneity across studies, with heterogeneity assessed using the *I*^2^ statistic. A subgroup analysis was conducted based on the dosage employed to explore the impact of dosage differences on study outcomes. Additionally, sensitivity analyses were performed to assess the robustness of the findings and determine the impact of individual studies on the overall results. Publication bias was assessed using funnel plot visualization, and Egger’s regression test. All statistical analyses were performed using STATA (Version 16.1; STATA Corporation LP, College Station, TX, USA), with statistical significance set at a *p*-value of less than 0.05.

## 3. Results

This systematic review and meta-analysis were registered with INPLASY, registration number INPLASY2024100031. The review process adhered to the PRISMA (Preferred Reporting Items for Systematic Reviews and Meta-Analyses) guidelines, ensuring a structured and transparent approach throughout the study.

### 3.1. Study Selection

**Figure 1:**
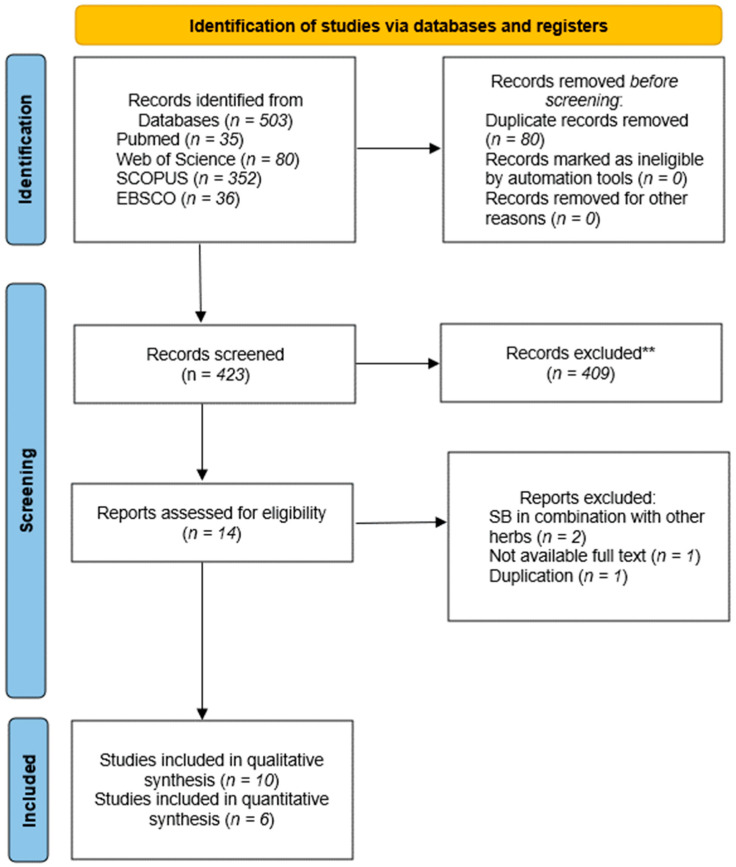
Flow chart. ** The articles were excluded based on the title and abstract review.

The flow diagram outlining the study selection process is depicted in Figure 1. Our comprehensive search initially identified a total of 503 records across various databases (PubMed: 35, Web of Science: 80, SCOPUS: 352, EBSCO: 36). After removing 80 duplicates, 423 records were screened based on their titles and abstracts against our inclusion criteria. Of these, 409 were excluded for not meeting the criteria, such as irrelevance to the study topic or lack of specific focus on SB. Subsequently, 14 full-text articles were assessed for eligibility. Four studies were excluded for reasons including the use of SB in combination with other herbs (*n* = 2) [21,22], lack of availability of full text (*n* = 1) [23], and duplication [24]. Ultimately, 10 studies were included for qualitative synthesis [11,25,26,27,28,29,30,31,32,33], with 6 studies qualifying for inclusion in the quantitative synthesis of the meta-analysis [25,27,30,31,32,33]. Studies such as those by Mabasa et al. [28], and Ngueguim et al. [11] were not included in the meta-analysis because the data were not entirely visible or well-defined in the graphs, making it impossible to extract the values using the AutoMeris WebPlotDigitizer software (version 5.2 for Windows 10).

### 3.2. Characteristics of the Study

Characteristics of the included studies are displayed in Table 1. The studies included were experimental in design and provided data on blood glucose (BG), insulin, Homeostatic Model Assessment of Insulin Resistance (HOMA-IR), total cholesterol (TC), triglycerides (TG), and various oxidative stress markers, such as malondialdehyde (MDA) and superoxide dismutase (SOD). A total of nine studies were performed on rats [11,25,26,27,28,30,31,32,33], with one study involving both rats and rabbits [29], and one study involving genetically modified mice [28]. Only one study included female animals [33], two studies included both male and female animals [26,29], and the remaining focused exclusively on male subjects.

**Figure 2:**
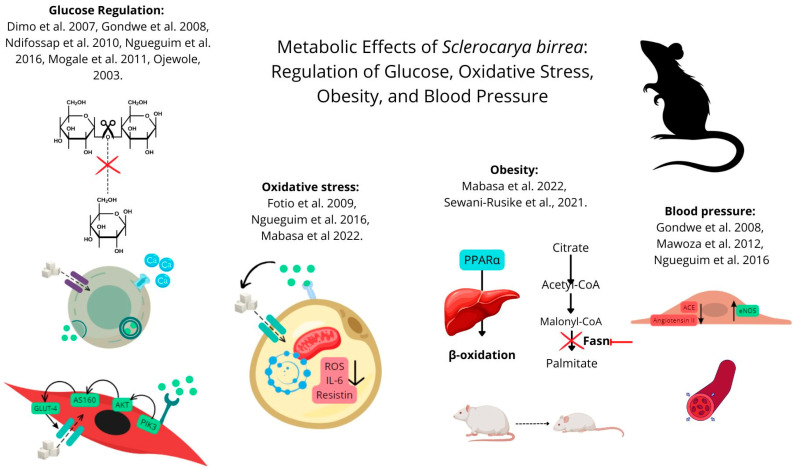
Hypothesized metabolic effects of SB in animal models [11,26,27,28,29,30,31,32,33,34]. SB may affect metabolic health through various pathways. In glucose metabolism, SB could enhance GLUT-4 translocation in muscle cells and promote insulin secretion in pancreatic β-cells by increasing calcium influx. For cardiovascular health, SB may support vasodilation and blood pressure regulation by increasing nitric oxide production via eNOS activation and inhibiting ACE activity. Additionally, SB may reduce inflammation by lowering pro-inflammatory cytokines like IL-6 and resistin in adipocytes, as well as decrease oxidative stress through its antioxidant effects. In the liver, SB might inhibit gluconeogenesis via AMPK activation, contributing to improved glycemic control.

The studies exhibited significant variability in the duration of the interventions, ranging from acute treatments to chronic interventions lasting up to 21 weeks. The primary intervention used was extracts from different parts of SB, including stem bark, fruit peel, and leaves. Dosages varied widely, from 60 mg/kg to 800 mg/kg per day, administered primarily via oral gavage. The age of the animals at the start of the experiments ranged from young adults to more mature specimens, with sample sizes typically ranging from 6 to 12 animals per experimental group. Table 1 and Figure 2 show the characteristics of the studies included in this systematic review with meta-analysis.

#### 3.2.1. Effect of SB on Lipid Profile

Several studies have investigated the effect of SB on the lipid profile [11,25,28,33], showing promising results in the modulation of lipid parameters. Dimo et al. [25] reported that SB stem bark methylene chloride/methanol extract significantly reduced plasma cholesterol and triglyceride levels in diabetic rats, particularly at higher doses. Similarly, Ngueguim et al. [11] found that SB stem bark aqueous extract decreased total cholesterol levels and abdominal fat in rats fed a high-fat diet, suggesting a beneficial role in managing lipid accumulation. Sewani-Rusike et al. [33] showed that SB fruit peel extract led to a reduction in total cholesterol and visceral fat in rats with diet-induced obesity, although triglycerides and LDL cholesterol levels remained elevated, indicating a selective lipid-lowering effect. Mabasa et al. [28] noted a reduction in liver weight and hepatic steatosis in *db*/*db* mice treated with SB, along with a downregulation of *Fasn*, a gene involved in lipid synthesis, suggesting that SB may influence lipid metabolism at the genetic level. These studies collectively highlight the potential role of various SB extracts in modulating lipid profiles, particularly by reducing cholesterol and other lipid accumulations in different animal models of metabolic dysfunction.

#### 3.2.2. Effect of SB on Blood Pressure and Endothelium-Dependent Vasodilation

Two studies have assessed the effect of SB on blood pressure, demonstrating varied impacts [11,29,33]. Ngueguim et al. [11] observed that SB stem bark aqueous extract reduced blood pressure in rats fed a diet high in oxidized palm oil and sucrose, which was associated with improved insulin sensitivity and reduced oxidative stress. In contrast, Sewani-Rusike et al. [33] found that SB fruit peel extract did not significantly affect blood pressure in rats with diet-induced obesity, despite improvements in other metabolic parameters such as body weight and glucose tolerance. Additionally, Mawoza et al. [29] investigated the vascular effects of SB leaf aqueous extract and reported that it caused significant, concentration-dependent endothelium-dependent vasodilation in isolated rabbit aortic rings and rat portal veins. This vasodilatory effect was shown to involve calcium channel pathways, as it was reduced by verapamil, a calcium channel blocker.

#### 3.2.3. Effect of SB on Oxidative Stress and Antioxidant Profile

Three studies evaluated the effect of SB on oxidative stress and antioxidant parameters [11,26,33]. Fotio et al. [26] reported that both aqueous and methanol extracts of SB stem bark significantly increased glutathione (GSH) levels and decreased MDA levels, indicating a reduction in oxidative stress. Ngueguim et al. [11] similarly showed that the stem bark aqueous extract of SB decreased oxidative stress markers, including a reduction in MDA levels and an increase in antioxidant enzyme activities such as SOD and catalase in rats fed a high-fat diet. Furthermore, Sewani-Rusike et al. [33] highlighted that the fruit peel extract of SB not only reduced oxidative stress but also improved the overall antioxidant profile by enhancing total antioxidant capacity.

### 3.3. Risk of Bias of the Study

**Figure 3:**
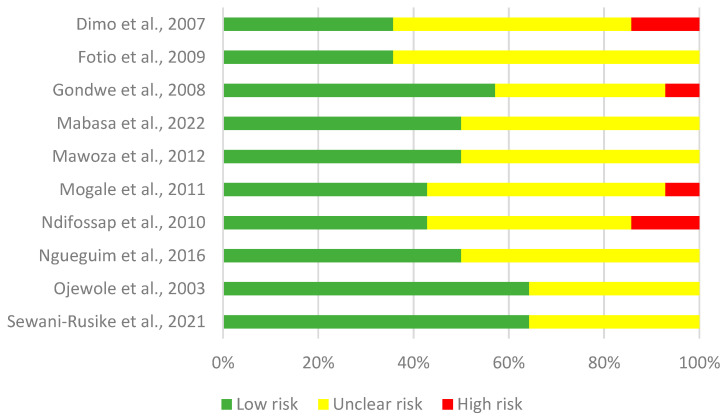
Risk of bias assessment across 10 studies [11,25,26,27,28,29,30,31,32,33].

The quality assessment of the 10 studies [11,25,26,27,28,29,30,31,32,33] was conducted using a tool adapted from SYRCLE’s risk of bias tool, developed by the SYRCLE group at Radboud University in Nijmegen, the Netherlands [20]. The results of the quality assessment of these studies are summarized in the attached Figure 3 and presented in detail in Table S1. On average, the studies scored 6.9 out of a possible 14 points (49.3%), with individual scores ranging from a minimum of 5 points (14.3%, observed in Dimo et al., [25] and Fotio et al. [26]) to a maximum of 9 points (14.3%, observed in Ojewole et al., [32] and Sewani-Rusike et al., [33]). Domains frequently lacking clarity included allocation concealment, blinding of outcome assessors, and random outcome assessment. Studies with a low risk of bias were predominant, representing a greater proportion (60%; [11,27,28,29,32,33]), while 40% of the studies had at least one domain with a high risk of bias [25,27,30,31], which may affect the validity of the obtained results. Overall, the lack of clear information in certain domains highlights the need to improve transparency in reported methodologies.

### 3.4. Meta-Analysis

#### 3.4.1. Acute Effects of SB on Blood Glucose Levels

**Figure 4:**
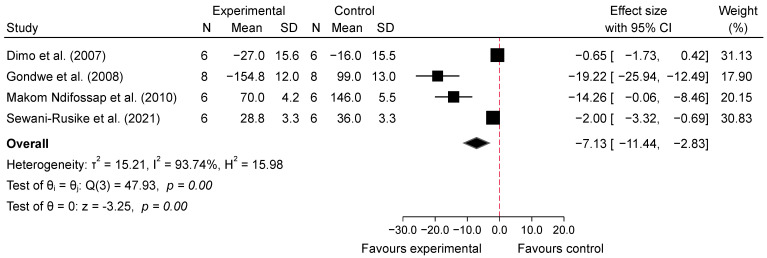
Forest plot of standardized mean difference (SMD) and 95% confidence interval (CI) in blood glucose (BG) between intervention and diabetes comparison group, following the consumption of low-dose SB at 1 h post-glucose feeding [25,27,31,33].

**Figure 5:**
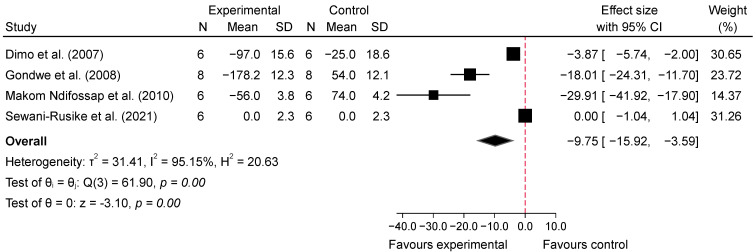
Forest plot of standardized mean difference (SMD) and 95% confidence interval (CI) in blood glucose (BG) between intervention and diabetes comparison group, following the consumption of low-dose SB at 2–4 h post-glucose feeding [25,27,31,33].

**Figure 6:**
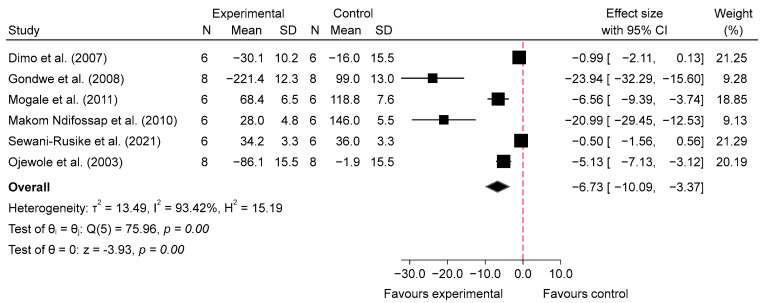
Forest plot of standardized mean difference (SMD) and 95% confidence interval (CI) in blood glucose (BG) between intervention and diabetes comparison group, following the consumption of high-dose SB at 1 h post-glucose feeding [25,27,30,31,32,33].

**Figure 7:**
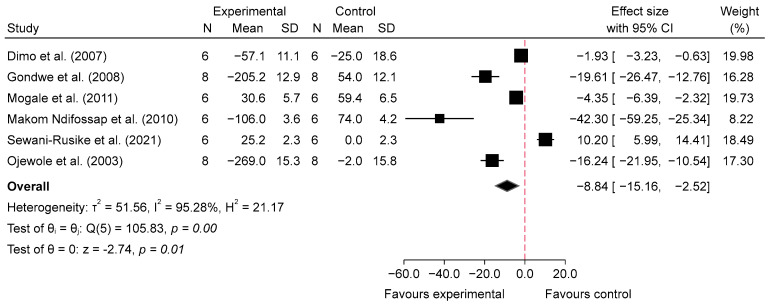
Forest plot of standardized mean difference (SMD) and 95% confidence interval (CI) in blood glucose (BG) between intervention and diabetes comparison group, following the consumption of high-dose SB at 2–4 h post-glucose feeding [25,27,30,31,32,33].

The meta-analysis evaluating the acute effects of SB on blood glucose levels in diabetic mice indicated a significant reduction in glucose levels in the intervention groups compared with the diabetic control groups. For low doses (100–150 mg/kg), the analysis of four studies [25,27,31,33] at 1 h post-administration showed a significant reduction in blood glucose levels, with an overall pooled standardized mean difference (SMD) of −7.13 (95% CI: −11.44 to −2.83, *p* = 0.00, *I*^2^ = 93.74%; Figure 4), indicating substantial glucose-lowering effects despite high heterogeneity. At 2–4 h post-administration, the glucose-lowering effect persisted, with a pooled SMD of −9.75 (95% CI: −15.92 to −3.59, *p* = 0.00, *I*^2^ = 95.15%; Figure 5), suggesting sustained efficacy of low doses over time, though high variability among studies was noted. In contrast, high doses (200–800 mg/kg) of SB also resulted in significant reductions in blood glucose levels. At 1 h post-administration, the analysis of six studies [25,27,30,31,32,33] showed a pooled SMD of −6.73 (95% CI: −10.09 to −3.37, *p* = 0.00, *I*^2^ = 93.42%; Figure 6), demonstrating a pronounced acute hypoglycemic effect with considerable heterogeneity. This effect was similarly sustained at 2–4 h post-administration, with a pooled SMD of −8.84 (95% CI: −15.16 to −2.52, *p* = 0.01, *I*^2^ = 95.28%; Figure 7). Additionally, comparisons between low and high doses revealed that at 1 h, high doses resulted in a greater reduction in blood glucose levels, with a pooled SMD of 2.76 (95% CI: −0.56 to 6.08, *p* = 0.10, *I*^2^ = 94.11%; Figure S1), indicating a trend favoring higher doses, although not statistically significant. At 2–4 h, the comparison between low and high doses showed a pooled SMD of 0.15 (95% CI: −5.03 to 5.33, *p* = 0.95, *I*^2^ = 95.69%; Figure S2), suggesting no significant difference between the doses, with high heterogeneity observed.

Subgroup analyses show that both low and high doses were effective in reducing BG at 1 h and 2–4 h post-glucose feeding, with similar effect sizes between the two dose levels. When comparing SB consumption (including both doses) with the diabetic control group at 1 h post-glucose feeding, the overall effect size was −6.42 (95% CI: −8.76 to −4.08; *p* = 0.00). For the low dose, the effect size was −7.13 (95% CI: −11.44 to −2.83; *I*^2^ = 93.74%), while for the 1 h high dose group, the effect size was −6.73 (95% CI: −10.09 to −3.37; *I*^2^ = 93.42%), also showing a significant reduction. Subgroup analysis showed no significant differences (*p* = 0.88), indicating that the treatment effect was similar between the two dose groups. These results can be found in Figure S3A of Supplementary Material.

At 2–4 h post-intervention, both low and high doses resulted in significant reductions in blood glucose levels. The low-dose group showed an effect size of −9.75 (95% CI: −15.92 to −3.59), while the high-dose group had an effect size of −8.84 (95% CI: −15.16 to −2.52). No significant difference was observed between doses (*p* = 0.84), suggesting comparable efficacy. These findings are presented in Figure S3B of the Supplementary Material.

Sensitivity analyses were performed to assess the robustness of the results by excluding each study individually, as shown in Figure S4. In Figure S4A, representing the sensitivity analysis for the low dose of SB at 1 h, the overall effect size remained stable at around −7.13; however, when the studies by Dimo et al. [25] or Sewani-Rusike et al. [33] were excluded, statistical significance was lost, suggesting that these studies may contribute significantly to the observed effect. In Figure S4B, the sensitivity analysis for the low dose of SB at 2–4 h post-administration showed no change after individual study exclusions, suggesting stability in the observed effect. For the SB high dose at 1 h (Figure S4C), the results of the sensitivity analysis remain stable despite the exclusion of individual studies. Finally, in Figure S4D (sensitivity analysis for the SB high dose at 2–4 h), an effect size close to −8.84 was observed. This statistical significance was lost when the study by Mogale et al. [30] was excluded, which may indicate that this study also reported a stronger effect, thus influencing the overall significance in this subgroup.

#### 3.4.2. Chronic Effects of SB on Blood Glucose Levels

**Figure 8:**
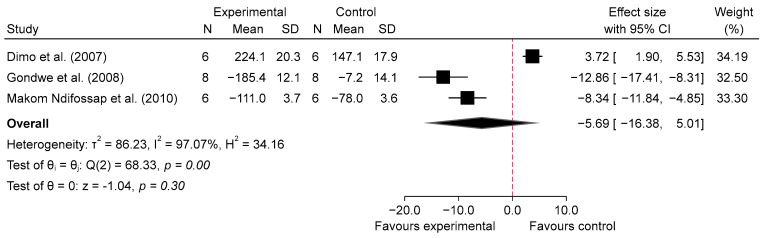
Forest plot of standardized mean difference (SMD) and 95% confidence interval (CI) in blood glucose (BG) between intervention and diabetes comparison group, following chronic consumption of SB at 5–6 weeks during fasting glucose [25,27,31].

In this case, meta-analysis was conducted for the chronic effects of SB using low doses [25,27,31], as specified in the methodology, meta-analyses were only performed when there were three or more studies with usable data. This analysis, focusing on the chronic administration of SB over 5 to 6 weeks at doses ranging from 120 to 150 mg/kg, revealed a non-significant overall effect on blood glucose levels in diabetic rats. The overall pooled SMD was −5.69 (95% CI: −16.38 to 5.01, *p* = 0.30, *I*^2^ = 97.07%; Figure 8), indicating no statistically significant reduction in blood glucose levels compared to the diabetic control group. The high heterogeneity observed (*I*^2^ = 97.07%) suggests substantial variability among the included studies, which may be due to differences in experimental designs, dosage regimens, or biological responses.

A sensitivity analysis for chronic SB consumption at 5–6 weeks during glucose fasting was performed to assess the robustness of the results by excluding each study individually (Figure S5). When the Dimo et al. [25] study was excluded, a statistically significant effect emerged, with an effect size of −10.36 (95% CI −14.75 to −5.96). This might suggest that the study by Dimo et al. may be attenuating the overall effect in the analysis of chronic SB consumption.

### 3.5. Publication Bias

The risk of publication bias was assessed through visual inspection of funnel plots and the application of Egger’s regression test for each comparison group. A total of seven funnel plots were generated to evaluate the relationship between study effect sizes and their standard errors in the following comparisons: low-dose SB vs. diabetic control (1 h post-administration; Figure S6A), low-dose SB vs. diabetic control (2–4 h post-administration; Figure S6B), high-dose SB vs. diabetic control (1 h post-administration; Figure S7A), high-dose SB vs. diabetic control (2–4 h post-administration; Figure S7B), high-dose SB vs. low-dose SB (1 h post-administration; Figure S8A), high-dose SB vs. low-dose SB (2–4 h post-administration; Figure S8B), and low-dose SB vs. diabetic control for chronic administration (Figure S9). All the figures mentioned can be found in the Supplementary Material.

Visual inspection of the funnel plots suggested asymmetry across all comparisons. To verify these results, Egger’s regression test was applied to each group. The results revealed statistically significant evidence of publication bias in most comparisons (*p* < 0.001), except for the high-dose vs. low-dose comparison in the 2–4 h post-administration period, where the test result showed no significant bias (*p* = 0.53).

## 4. Discussion

This study aimed to systematically review and meta-analyze the effects of SB on glycemic control and various metabolic parameters in diabetic animal models, offering insights into its potential therapeutic role. Our comprehensive analysis included acute and chronic interventions, focusing on the modulation of blood glucose levels, lipid profiles, blood pressure, and oxidative stress. Through this research, we aimed to hypothesize how SB might exert its effects based on the available literature and evaluate its suitability as a complementary treatment in managing diabetes and associated metabolic disorders. The use of nutraceuticals for managing these metabolic parameters has gained increasing attention in recent years [34]. Several meta-analyses and systematic reviews have suggested the beneficial effects of various nutraceuticals. For instance, a meta-analysis on *Hibiscus sabdariffa* indicated significant reductions in both systolic and diastolic blood pressure, highlighting its potential role in hypertension management [35]. Berberine has been systematically reviewed for its impact on glycemic control, showing potential reductions in fasting blood glucose and HbA1c levels [36]. Another meta-analysis on *Curcuma longa* (turmeric) found that curcumin supplementation may improve markers of oxidative stress and increase antioxidant capacity, suggesting its potential role in mitigating diabetes-related complications [37].

Additionally, a systematic review of green tea catechins reported reductions in total cholesterol and LDL cholesterol, providing evidence for its cardiovascular protective effects [38]. A meta-analysis of *Nigella sativa* (black seed) focused on its lipid-lowering effects, showing significant reductions in total cholesterol, LDL cholesterol, and triglycerides, which may offer cardioprotective benefits [39]. Similarly, *Elettaria cardamomum* (cardamom) has been reviewed for its lipid-lowering and antioxidant properties, with findings suggesting positive effects on serum lipid profiles [40]. Furthermore, *Cinnamomum verum* (cinnamon) was found in a meta-analysis to possibly improve fasting blood glucose levels and insulin sensitivity, underscoring its potential utility in managing diabetes [41]. These findings collectively support the potential of nutraceuticals, including SB, as viable options for managing glycemic control, lipid profiles, blood pressure, and oxidative stress, advocating for their integration into holistic management strategies for metabolic disorders.

### 4.1. Carbohydrate Metabolism and Potential Hypoglycemic Mechanisms

Our meta-analysis suggests that SB may significantly reduce blood glucose levels in diabetic animal models, both acutely and chronically. The analysis of acute effects indicated that low doses (100–150 mg/kg) of SB were associated with a substantial reduction in blood glucose levels at 1 h post-administration (SMD = −7.13, 95% CI: −11.44 to −2.83, *p* = 0.00, *I*^2^ = 93.74%) and 2–4 h post-administration (SMD = −9.75, 95% CI: −15.92 to −3.59, *p* = 0.00, *I*^2^ = 95.15%). High doses (200–800 mg/kg) also showed significant reductions at 1 h (SMD = −6.73, 95% CI: −10.09 to −3.37, *p* = 0.00, *I*^2^ = 93.42%) and at 2–4 h (SMD = −8.84, 95% CI: −15.16 to −2.52, *p* = 0.01, *I*^2^ = 95.28%). Interestingly, while high doses resulted in a greater reduction at 1 h compared to low doses (SMD = 2.76, 95% CI: −0.56 to 6.08, *p* = 0.10, *I*^2^ = 94.11%), this trend was not statistically significant, and no significant difference was observed at 2–4 h (SMD = 0.15, 95% CI: −5.03 to 5.33, *p* = 0.95, *I*^2^ = 95.69%).

These results highlight the potential for SB to exert significant acute hypoglycemic effects, potentially through several mechanisms. One plausible pathway is the activation of AMPK, like the action of metformin [42]. AMPK activation may enhance glucose uptake and utilization while inhibiting hepatic gluconeogenesis, thus contributing to improved glycemic control [43]. The compounds in SB, such as epicatechin (EC) and epigallocatechin gallate (EGCG) [44], are known to interact with these pathways, suggesting that SB may enhance insulin sensitivity and promote glucose uptake in peripheral tissues [45,46].

An important aspect to consider is the difference in the impact observed at 1 h versus 2–4 h post-administration, which may reflect distinct physiological processes. The significant reduction in blood glucose levels at 1 h is likely related to the inhibition of hepatic gluconeogenesis [47]. This process is crucial in maintaining fasting glucose levels and is a primary target for AMPK activation. In contrast, the sustained reduction observed at 2–4 h post-administration suggests enhanced peripheral insulin sensitivity, particularly in skeletal muscle [48]. This time-dependent effect could be due to the increased translocation of GLUT-4 transporters to the muscle cell membrane, facilitating greater glucose uptake and utilization. Thus, the early effect of SB may be attributed to its action on the liver, whereas the latter effect aligns more with improving insulin signaling pathways in peripheral tissues. Understanding these temporal differences is crucial, as they may indicate SB’s dual role in addressing both hepatic insulin resistance and peripheral glucose uptake, which are differently implicated in conditions such as impaired fasting glucose (IFG) and impaired glucose tolerance (IGT) [47,48].

SB might also inhibit the activities of α-amylase and α-glucosidase, enzymes involved in the digestion of carbohydrates [49]. By reducing the enzymatic breakdown of complex carbohydrates into glucose, SB could lower the rate of glucose absorption in the intestines, resulting in reduced postprandial glucose spikes [50]. This mechanism could be particularly relevant in managing postprandial hyperglycemia, a common issue in diabetes management

Furthermore, SB may influence glucose homeostasis by enhancing insulin signaling pathways [51]. SB might improve the sensitivity of insulin receptors, which would facilitate glucose uptake by muscle and adipose tissues [51]. The presence of flavonoids in SB might modulate the phosphorylation of key proteins in the insulin signaling cascade, such as insulin receptor substrate (IRS) and phosphoinositide 3-kinase (PI3K), further promoting glucose uptake and utilization [52].

Additionally, SB may have a direct effect on pancreatic β-cells, promoting insulin secretion. Studies have suggested that SB could enhance glucose-stimulated insulin secretion by increasing intracellular calcium levels, which are essential for insulin vesicle fusion with the cell membrane [31]. This increased calcium influx could trigger insulin release, aiding in maintaining adequate insulin levels in diabetic conditions and thus contributing to better glycemic control [53].

Borochov-Neori et al. [54] investigated the effects of SB juice consumption on healthy human subjects and found no significant changes in fasting blood glucose levels. This finding indicates that while SB may have strong hypoglycemic effects in animal models, its impact on blood glucose in non-diabetic humans may be limited. These results align with those of Victoria-Montesinos et al. [55], suggesting that SB’s hypoglycemic effects might be more pronounced in individuals with impaired glucose regulation or those already exhibiting hyperglycemia, rather than in normoglycemic individuals. This distinction emphasizes the need for targeted use of SB in populations at risk for or suffering from hyperglycemia and diabetes.

It is important to note that the heterogeneity observed in the results of our meta-analysis could be attributed to the limited number of studies available for inclusion. This variability might also arise from differences in experimental design, including variations in dosage, duration of treatment, and animal models used.

### 4.2. Lipid Metabolism: Potential Effects on Cholesterol and Triglycerides

Beyond its role in glucose metabolism, SB may exert beneficial effects on lipid metabolism, offering potential cardioprotective properties. The phytochemical composition of SB, rich in polyphenols, fatty acids, and phytosterols, suggests multiple mechanisms through which SB could impact lipid profiles. Studies included in this review indicated that SB extracts might reduce total cholesterol, LDL cholesterol, and triglycerides [11,25,28]. One of the key mechanisms might involve the inhibition of intestinal cholesterol absorption, mediated by the phytosterols present in SB [56]. These phytosterols compete with dietary cholesterol for absorption sites in the intestines, effectively reducing the amount of cholesterol entering the bloodstream [57].

Moreover, SB might influence lipid metabolism at the cellular level by activating peroxisome proliferator-activated receptor alpha (PPARα) [28], a nuclear receptor that regulates genes involved in lipid metabolism [58]. Activation of PPARα can enhance the oxidation of fatty acids, reduce triglyceride synthesis, and improve lipid profiles [59]. The fatty acid composition of SB, particularly its oleic and linoleic acids [60], might play a role in this activation, leading to reduced plasma triglyceride levels and improved lipid handling [61].

Borochov-Neori et al. [54] found that consumption of SB juice over a period of three weeks led to a significant reduction in total cholesterol and LDL cholesterol levels in healthy subjects, along with an increase in HDL cholesterol levels. These findings corroborate those of Victoria-Montesinos et al. [55], showing that SB can positively influence lipid profiles in both healthy and prediabetic individuals. The reduction in LDL cholesterol and increase in HDL cholesterol suggest that SB may enhance cholesterol transport and clearance from the bloodstream, which could help in reducing the risk of atherosclerosis and cardiovascular diseases [62]. These effects are likely mediated by the phytosterols, and polyphenolic compounds present in SB, which interfere with cholesterol absorption and metabolism [63].

### 4.3. Blood Pressure and Endothelial Function: Role of Nitric Oxide Synthase

The beneficial effects of SB on cardiovascular health may extend to blood pressure regulation and endothelial function [49]. The potential of SB to enhance endothelium-dependent vasodilation is linked to its effects on nitric oxide (NO) production via endothelial nitric oxide synthase (eNOS) [29]. NO is a crucial vasodilator that plays a key role in maintaining vascular tone by relaxing blood vessels and reducing blood pressure [64]. The flavonoid components of SB might enhance eNOS activity or expression, leading to an increase in NO availability [29]. This could counteract the effects of oxidative stress, which often reduces NO bioavailability by increasing the production of reactive oxygen species (ROS) that degrade NO [65].

Additionally, SB might modulate blood pressure by influencing the renin-angiotensin system (RAS) [66]. By inhibiting angiotensin-converting enzyme (ACE), SB could reduce the production of angiotensin II, a potent vasoconstrictor, thereby lowering blood pressure [67]. The polyphenolic compounds in SB may inhibit ACE activity, like the mechanism of action observed in ACE inhibitors used to treat hypertension [68]. This dual mechanism—enhancing NO production and inhibiting ACE—could provide a comprehensive approach to managing hypertension and improving vascular health.

Victoria-Montesinos et al. [55] also observed significant improvements in endothelial function, as indicated by increased flow-mediated dilation (FMD) in prediabetic subjects after chronic SB extract consumption. However, no significant changes in blood pressure were noted, which could suggest that while SB may improve endothelial function, its effects on blood pressure might require longer treatment duration or higher doses to become apparent.

### 4.4. Antioxidant and Anti-Inflammatory Properties

The antioxidant properties of SB are another critical component of its potential therapeutic effects [69]. The presence of flavonoids and other polyphenolic compounds in SB [8] suggests that it could effectively scavenge ROS, thereby reducing oxidative stress [14]. This reduction in oxidative stress markers, such as MDA, and the enhancement of antioxidant enzymes like SOD, highlight SB’s potential role in protecting against oxidative damage, which is a significant factor in the development of diabetic complications [70].

**Figure 9:**
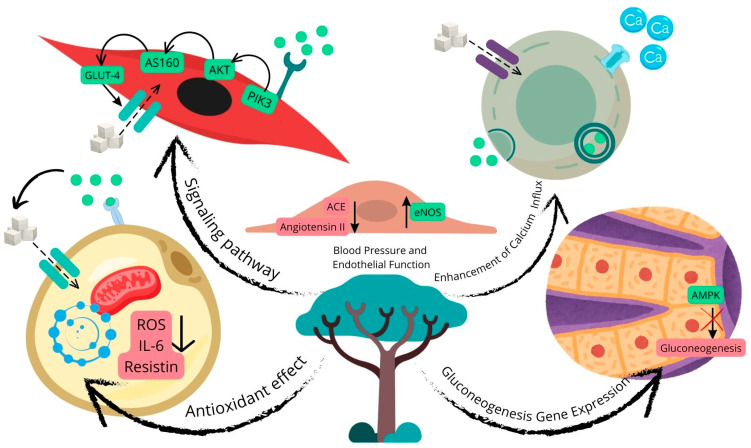
Possible mechanisms of action of SB. This image hypothesizes the potential mechanisms by which SB may influence various metabolic processes. In carbohydrate metabolism, SB could enhance glucose uptake in muscle cells by facilitating the translocation of glucose transporter type 4 (GLUT-4) to the cell membrane. This might be mediated by activating signaling pathways involving proteins such as AS160, AKT, and phosphoinositide 3-kinase (PI3K), which play roles in the insulin signaling cascade. Through these pathways, SB may improve insulin sensitivity and facilitate glucose entry into cells, helping to reduce blood glucose levels. In pancreatic β-cells, SB might promote insulin secretion by increasing intracellular calcium influx. Elevated calcium levels are crucial for the fusion of insulin vesicles with the cell membrane, potentially enhancing insulin release and maintaining adequate insulin levels, especially under diabetic conditions. Regarding lipid metabolism and cardiovascular health, SB may affect blood pressure regulation and endothelial function by potentially increasing endothelial nitric oxide synthase (eNOS) activity, leading to enhanced nitric oxide (NO) production. NO is a key vasodilator, promoting vascular relaxation and reducing blood pressure. Additionally, SB could inhibit the angiotensin-converting enzyme (ACE), which converts angiotensin I into the vasoconstrictor angiotensin II. By inhibiting ACE, SB may promote vasodilation and help manage hypertension. This dual action—enhancing eNOS activity and inhibiting ACE—suggests that SB could play a role in modulating blood pressure and supporting vascular health. In adipocytes, SB might reduce the production of pro-inflammatory cytokines such as interleukin-6 (IL-6) and resistin, both linked to inflammation and insulin resistance. By lowering their levels, SB could reduce inflammation and enhance insulin sensitivity, contributing to its antidiabetic effects. SB’s potential antioxidative properties might also help reduce the production of reactive oxygen species (ROS), which contribute to oxidative stress and inflammation in metabolic disorders. Finally, in the liver, SB may inhibit the expression of genes involved in gluconeogenesis, potentially through the activation of AMP-activated protein kinase (AMPK). By suppressing gluconeogenic pathways, SB could reduce hepatic glucose output, aiding in overall glycemic control. These hypothesized pathways illustrate the possible therapeutic roles of SB in managing elements of metabolic syndrome, such as hyperglycemia, hypertension, and oxidative stress, suggesting its potential as a complementary therapy for metabolic disorders.

SB’s potential anti-inflammatory effects may also contribute to its role in managing diabetes and cardiovascular diseases [71]. By inhibiting pro-inflammatory cytokines such as interleukin-6 (IL-6) and tumor necrosis factor-alpha (TNF-α) [72], SB could reduce chronic inflammation [73], a key factor in insulin resistance and atherosclerosis [74]. The anti-inflammatory actions of SB could be mediated through the inhibition of enzymes involved in the inflammatory response, such as cyclooxygenase (COX) and lipoxygenase (LOX) [71]. These enzymes play a critical role in the synthesis of pro-inflammatory mediators [75]; thus, their inhibition could lead to reduced inflammation and improved metabolic health. Figure 9 summarizes the possible mechanisms of action that SB may have.

### 4.5. Limitations

The present review and meta-analysis have several strengths and limitations. A major strength of this study is that, to the best of our knowledge, it represents the first systematic review and meta-analysis specifically assessing the effects of SB on diabetes and metabolic parameters in experimental animal models. A comprehensive search strategy was employed, utilizing leading databases such as PubMed, Web of Science, SCOPUS, and EBSCO to ensure an exhaustive identification of relevant studies. The inclusion of only in vivo animal studies likely reduces the risk of selection bias, providing a focused perspective on the biological effects of SB. Additionally, sensitivity analyses were conducted to evaluate the robustness of the meta-analysis findings, lending further reliability to the results.

However, there are notable limitations to this study. The high degree of heterogeneity observed among the included studies may be attributed to variations in study design, types of diabetes-induced, intervention types, dosages, and duration of the treatments. This variability, which was also evident in the subgroup analyses, suggests that the pooled estimates should be interpreted with caution. Furthermore, many of the included studies lacked detailed reporting on key methodological aspects such as randomization, allocation concealment, and blinding of outcome assessors, which could introduce bias. The absence of blinding in most experimental setups increases the risk of performance bias, as both participants and researchers might influence the outcomes.

Our exclusion criteria focused on ensuring the relevance and quality of the data included. Specifically, studies that were in vitro, clinical trials, review articles, case reports, letters, abstracts from conferences, or those examining the effects of SB in combination with other plants were excluded. This approach aimed to maintain a high level of specificity regarding the effects of SB alone. However, this also limited the number of studies eligible for inclusion, which may have impacted the diversity and breadth of the findings. In this regard, although the number of studies included in this meta-analysis could be considered scarce, it aligns with previous publications that have also explored nutritional aspects and health effects in humans or animal models. For example, similar meta-analyses have included a limited number of studies, such as the work by Morvaridzadeh et al. [76] on the effect of cashew nut on lipid profile and Montgomery et al. [77] on S-adenosylmethionine and cognitive performance in mice, both of which synthesized data from relatively small samples (3–7 studies). Furthermore, the study by Sahebkar et al. [78] on curcuminoids and oxidative stress provides additional support for the feasibility of meta-analyses with small sample sizes in nutraceutical research, where available data are often limited.

Another potential limitation is the relatively small number of studies that met the inclusion criteria for meta-analysis, which restricts the generalizability of the results. Despite an extensive search strategy, there may still be incomplete retrieval of published documents, which could impact the overall conclusions drawn from this study.

## 5. Conclusions

SB shows promising potential as a nutraceutical agent in the management of metabolic disorders, particularly in reducing blood glucose levels and improving other cardiometabolic risk parameters, such as lipid profiles and oxidative stress markers. The results of this systematic review and meta-analysis suggest that the bioactive compounds present in SB, especially polyphenols, may exert beneficial effects on glucose homeostasis, thereby contributing to the prevention and management of diseases like diabetes and cardiovascular disorders.

However, the variability observed in the study outcomes highlights the need for further research to confirm these effects. It is essential to conduct more standardized animal studies, as well as well-designed clinical trials in humans, to validate the efficacy of SB and to better understand the mechanisms underlying its therapeutic effects.

## Figures and Tables

**Table 1:** Study characteristics.

Authors (Year)	Species/Strain	Sex	DM Inducing Agent	Duration of Intervention	Type of Intervention	Dose (Route)	Outcome	Additional Parameters	Organ/System Affected	Control/Comparison Group	Conclusions
Dimo et al. (2007) [25]	Male Wistar rats	Male	STZ	Acute and chronic (up to 21 days)	Stem bark methylene chloride/methanol extract of SB	150 mg/kg and 300 mg/kg, oral administration	Significant reduction in blood glucose, increased plasma insulin levels, prevention of body weight loss, improved glucose tolerance	Reduced plasma cholesterol, triglyceride, and urea levels at 300 mg/kg dose, a significant decrease in food and fluid intake, improved oral glucose tolerance test	Blood glucose regulation, insulin secretion, body weight, plasma cholesterol, triglycerides, urea levels	Diabetic rats treated with distilled water; diabetic rats treated with metformin (500 mg/kg)	SB extract improves glucose homeostasis in STZ-induced diabetic rats, possibly by stimulating insulin secretion and reducing glucose absorption
Fotio et al. (2009) [26]	Wistar rats	Male and Female	None	Acute and chronic (up to 21 days)	Stem bark aqueous and methanol extracts of SB	150 mg/kg and 300 mg/kg, oral administration	Significant inhibition of paw edema, reduction in nitrite levels, increase in GSH levels, decrease in MDA levels	Evaluated on carrageenan-, histamine-, serotonin-induced acute inflammation; formalin- and CFA-induced chronic inflammation	Inflammation (paw edema), oxidative stress	Rats treated with distilled water, rats treated with indomethacin, diclofenac, dexamethasone	SB extracts exhibit significant anti-inflammatory activity by inhibiting histamine and prostaglandin pathways and exhibiting antioxidant properties
Gondwe et al. (2008) [27]	Wistar rats	Male	STZ	Acute and chronic (up to 5 weeks)	Stem bark ethanolic extract of SB	60, 120, 240 mg/kg, orally	Dose-dependent reduction in blood glucose; decreased plasma urea and creatinine levels; increased GFR; reduced MAP	Studied acute and chronic effects; evaluated effects on renal function, mean arterial blood pressure, and insulin levels	Blood glucose, renal function, MAP	Rats treated with deionized water, insulin, metformin, glibenclamide, propranolol	SB shows potential hypoglycemic, renoprotective, and hypotensive effects, suggesting its use as a complementary remedy in diabetes management
Mabasa et al. (2022) [28]	db/db mice	NR	Genetic mutation (Lepr^db/db^)	4 weeks	MLE	600 mg/kg, orally	MLE reduced body weight and liver weight, decreased hepatic steatosis, downregulated Fasn, and upregulated Pparα and Cpt1. No significant effect on blood glucose.	Study included histological assessment, gene expression analysis (qRT-PCR), and protein expression analysis (Western blot).	Liver, hepatic steatosis	Obese control, metformin-treated group	MLE inhibits hepatic steatosis via activation of β-oxidation and reduction in lipogenesis, potentially influencing DNA methylation processes
Mawoza et al. (2012) [29]	Wistar rats, New Zealand white rabbits	Male and female	None	Acute	SB leaf aqueous extract	50–400 mg/mL	SB extract caused significant, concentration-dependent contractile effects on rabbit aortic rings and rat portal veins. Verapamil reduced these effects, indicating the involvement of calcium channels.	Investigated contractile effects with endothelium-intact/-denuded tissues, and involvement of COX and nitric oxide pathways using L-NAME and indomethacin.	Vascular smooth muscles (aortic rings, portal veins)	Endothelium-intact vs. endothelium-denuded aortic rings; presence vs. absence of inhibitors (verapamil, L-NAME, indomethacin)	SB extract may have spasmogenic effects, potentially increasing blood pressure. Contrary to its traditional use for hypertension, SB extract might induce or exacerbate hypertension
Mogale et al. (2011) [30]	Albino Wistar rats	Male	Alloxan monohydrate	Acute	SB stem bark extracts	300 mg/kg (oral)	SB hexane extract significantly inhibited α-glucosidase activity in vitro, comparable to acarbose, and suppressed postprandial hyperglycemia in vivo following sucrose load. It did not affect PPHG after starch or glucose load.	α-Amylase and α-glucosidase inhibition; tested with starch and sucrose tolerance tests.	Digestive system (intestinal enzymes)	Acarbose as a comparison for α-amylase and α-glucosidase inhibition. Normal and diabetic rats treated with water.	SB hexane extract may effectively inhibit α-glucosidase and suppress PPHG with fewer side effects compared to acarbose
Ndifossap et al. (2010) [31]	Wistar rats	Male	STZ, preceded by nicotinamide	Acute and chronic (up to 2 weeks)	SB stem-bark extract	150 mg/kg and 300 mg/kg (oral)	SB extract corrected hyperglycemia and restored plasma insulin levels. Enhanced glucose-stimulated insulin secretion in INS-1E cells and isolated rat islets after 24 h exposure.	Glucose tolerance test; insulin secretion assays; ATP generation; gene expression analysis.	Pancreatic β-cells	Non-diabetic and diabetic control groups; Glibenclamide-treated group	SB extract enhanced glucose metabolism and insulin secretion, suggesting potential for managing diabetes by acting on pancreatic β-cells
Ngueguim et al. (2016) [11]	Wistar rats	Male	oxidized palm oil (10%) and sucrose (10%)	Acute and chronic (16 weeks + 3 weeks intervention)	SB stem-bark aqueous extract	150 mg/kg and 300 mg/kg (oral)	Reduced hyperglycemia, improved glucose tolerance and insulin sensitivity, reduced body weight and abdominal fat, decreased oxidative stress and blood pressure	OGTT, insulin tolerance test, lipid profile, ALT and AST levels, SOD and catalase activity, nitrites, MDA levels	Liver, kidney, heart, aorta, and pancreas	Standard diet group, SOPO + S diet group, glibenclamide group	SB extract demonstrated hypoglycemic, antihyperlipidemic, antihypertensive, and antioxidant properties, supporting its traditional use in managing diabetes
Ojewole (2003) [32]	Wistar rats	Male	STZ	Acute treatment	SB stem-bark aqueous extract	100–800 mg/kg (oral)	Dose-dependent hypoglycemic effect, with significant reductions in blood glucose levels observed	Blood glucose measurements at different time intervals	Pancreas, blood glucose levels	Normal control rats, chlorpropamide (250 mg/kg)	SB extract exhibits hypoglycemic effects, supporting its traditional use in managing type 2 diabetes mellitus in African communities
Sewani-Rusike et al. (2021) [33]	Wistar rats	Female	HED	Acute and chronic (4 weeks after 15 weeks HED)	SB fruit peel extract	100 mg/kg BW and 200 mg/kg BW (oral)	Reduced body weight, visceral fat, total cholesterol, insulin, and HOMA-IR; improved glucose tolerance and reduced hepatic steatosis. Blood pressure, triglycerides, and LDL cholesterol levels remained high.	Fasting glucose, OGTT, blood pressure, visceral fat, liver lipids	Liver, adipose tissue	Normal diet control, HED control	SB fruit peel extract ameliorates diet-induced obesity and metabolic syndrome by reducing visceral fat, improving insulin resistance, inflammation, and NAFLD; stabilizing leptin: adiponectin balance

ALT: Alanine aminotransferase; AST: Aspartate aminotransferase; ATP: Adenosine triphosphate; BW: Body weight; CFA: Complete Freund’s adjuvant; COX: Cyclooxygenase; DM: Diabetes mellitus; GFR: Glomerular filtration rate; GSH: Glutathione; HED: High energy diet; HOMA-IR: Homeostatic model assessment of insulin resistance; L-NAME: Nω-Nitro-L-arginine methyl ester; Lepr^db/db^: Leptin receptor gene mutation in diabetic mice; LDL: Low-density lipoprotein; MDA: Malondialdehyde; MLE: Methanolic leaf extract; NAFLD: Non-alcoholic fatty liver disease; NR: Not reported; OGTT: Oral glucose tolerance test; PPHG: Postprandial hyperglycemia; Pparα: Peroxisome proliferator-activated receptor alpha; SB: *Sclerocarya birrea*; SOPO: Oxidized palm oil; SOD: Superoxide dismutase; STZ: Streptozotocin.

## Data Availability

Data are contained within the article.

## Supplementary Materials

The following supporting information can be downloaded at https://www.mdpi.com/article/10.3390/metabo14110615/s1. Figure S1: Forest plot of standardized mean difference (SMD) and 95% confidence interval (CI) in blood glucose (BG) between the consumption of low-dose and high-dose SB at 1 h post-glucose feeding. Figure S2: Forest plot of standardized mean difference (SMD) and 95% confidence interval (CI) in blood glucose (BG) between the consumption of low-dose and high-dose SB at 2–4 h post-glucose feeding. Figure S3: Forest plot of standardized mean difference (SMD) and 95% confidence interval (CI) in blood glucose (BG) for subgroup analysis between the intervention and diabetes comparison groups after SB consumption: (A) 1 h post-feeding; (B) 2–4 h post-feeding. Figure S4: Sensitivity analysis for blood glucose levels in acute consumption of SB. (A) Low dose, 1 h post-glucose feeding; (B) Low dose, 2–4 h post-glucose feeding; (C) High dose, 1 h post-glucose feeding; (D) High dose, 2–4 h post-glucose feeding. Figure S5: Sensitivity analysis for chronic SB consumption at 5–6 weeks fasting on blood glucose levels. Figure S6: Funnel Plot Low-dose SB vs. diabetic control. Figure S7: Funnel Plot High-dose SB vs. diabetic control. Figure S8: Funnel plot high-dose SB vs. low dose. Figure S9: Funnel Plot for the comparison of low-dose SB in chronic. Table S1: SYRCLE’s risk of bias tool; Table S2: Raw data for hyperglycemic control group; Table S3: Raw data for SB low dose; Table S4: Raw data for SB high dose; Table S5: Raw data for chronic consumption of SB.
