# Supplementary material for: Systematic Review and Meta-Analysis of Sclerocarya birrea on Metabolic Disorders: Evidence from Preclinical Studies
Source: Metabolites. 2024 Nov 12;14(11):615. doi: 10.3390/metabo14110615 (PMC11596559; doi:10.3390/metabo14110615)
Supplement: Supplementary file 1 [file metabolites-14-00615-s001.zip › Table S1.pdf]

Table S1: SYRCLE's risk of bias tool

Study	Selection bias			Performance bias		Detection bias		Attrition bias	Reporting bias	Other					Total score (on 14)
	**Sequence generation**	**Baseline characteristic**	**Allocation concealment**	**Random housing**	**Blinding**	**Random outcome assessment**	**Blinding**	**Incomplete outcome data**	**Free of selective outcome reporting**	**Free of contamination**	**Free of inappropriate influence of funders**	**Free of unite of analysis errors**	**Design specific risk of bias**	**New animals added to replace drop-outs**	
Dimo et al., 2007	Unclear	Low	Unclear	Unclear	High	Unclear	High	Unclear	Low	Low	Low	Low	Unclear	Unclear	5
Fotio et al., 2009	Unclear	Low	Unclear	Unclear	Unclear	Unclear	Unclear	Unclear	Low	Low	Low	Low	Unclear	Unclear	5
Gondwe et al., 2008	Low	Low	Unclear	Low	Unclear	Unclear	High	Low	Low	Low	Low	Low	Unclear	Unclear	8
Mabasa et al., 2022	Low	Low	Unclear	Unclear	Unclear	Unclear	Unclear	Low	Low	Low	Low	Low	Unclear	Unclear	7
Mawoza et al., 2012	Unclear	Unclear	Low	Unclear	Unclear	Low	Unclear	Low	Low	Low	Low	Low	Unclear	Unclear	7
Mogale et al., 2011	Unclear	Low	Unclear	Unclear	Unclear	Unclear	High	Low	Low	Low	Low	Low	Unclear	Unclear	6
Ndifossap et al., 2010	Unclear	Low	Unclear	Unclear	High	Unclear	High	Low	Low	Low	Low	Low	Unclear	Unclear	6
Ngueguim et al., 2016	Unclear	Low	Unclear	Unclear	Unclear	Unclear	Low	Low	Low	Low	Low	Low	Unclear	Unclear	7
Ojewole et al., 2003	Low	Low	Unclear	Low	Unclear	Unclear	Low	Low	Low	Low	Low	Low	Unclear	Unclear	9
Sewani-Rusike et al., 2021	Low	Low	Unclear	Low	Unclear	Unclear	Low	Low	Low	Low	Low	Low	Unclear	Unclear	9

1. Dimo, T.; Rakotonirina, S. V.; Tan, P. V.; Azay, J.; Dongo, E.; Kamtchouing, P.; Cros, G. Effect of *Sclerocarya birrea* (Anacardiaceae) Stem Bark Methylene Chloride/Methanol Extract on Streptozotocin-Diabetic Rats. *J. Ethnopharmacol.* **2007**, *110*, 434–438. https://doi.org/10.1016/j.jep.2006.10.020.
2. Fotio, A.L.; Dimo, T.; Nguelefack, T.B.; Dzeufiet, P.D.D.; Ngo Lemba, E.; Temdie, R.J.; Ngueguim, F.; Olleros, M.L.; Vesin, D.; Dongo, E.; et al. Acute and Chronic Anti-Inflammatory Properties of the Stem Bark Aqueous and Methanol Extracts of *Sclerocarya birrea* (Anacardiaceae). *Inflammopharmacology* **2009**, *17*, 229–237. https://doi.org/10.1007/s10787-009-0011-2.
3. Gondwe, M.; Kamadyaapa, D.R.; Tufts, M.; Chuturgoon, A.A.; Musabayane, C.T. *Sclerocarya birrea* [(A. Rich.) Hochst.] [Anacardiaceae] Stem-Bark Ethanolic Extract (SBE) Modulates Blood Glucose, Glomerular Filtration Rate (GFR) and Mean Arterial Blood Pressure (MAP) of STZ-Induced Diabetic Rats. *Phytomedicine* **2008**, *15*, 699–709. https://doi.org/10.1016/j.phymed.2008.02.004.

4. Mabasa, L.; Kotze, A.; Shabalala, S.; Kimani, C.; Gabuza, K.; Johnson, R.; Sangweni, N.F.; Maharaj, V.; Muller, C.J.F.; L, M.; et al. *Sclerocarya birrea* (Marula) Extract Inhibits Hepatic Steatosis in Db/Db Mice. *Int. J. Environ. Res. Public Health* **2022**, *19*, 3782. https://doi.org/10.3390/ijerph19073782.
5. Mawoza, T.; Ojewole, J.A.; Owira, P.M. Contractile Effect of *Sclerocarya birrea* (A Rich) Hochst (Anacardiaceae) (Marula) Leaf Aqueous Extract on Rat and Rabbit Isolated Vascular Smooth Muscles. *Cardiovasc. J. Afr.* **2012**, *23*, 12–17.
6. Mogale, M.A.; Lebelo, S.L.; Thovhogi, N.; de Freitas, A.N.; Shai, L.J. α-Amylase and α-Glucosidase Inhibitory Effects of *Sclerocarya birrea* [(A. Rich.) Hochst.] Subspecies *Caffra* (Sond) Kokwaro (Anacardiaceae) Stem-Bark Extracts. *Afr. J. Biotechnol.* **2011**, *10*, 15033–15039. https://doi.org/10.5897/AJB11.1408.
7. Makom Ndifossap, I.G.; Frigerio, F.; Casimir, M.; Ngueguim Tsofack, F.; Dongo, E.; Kamtchouing, P.; Dimo, T.T.; Maechler, P.; Ndifossap, I.G.M.; Frigerio, F.; et al. *Sclerocarya birrea* (Anacardiaceae) Stem-Bark Extract Corrects Glycaemia in Diabetic Rats and Acts on Beta-Cells by Enhancing Glucose-Stimulated Insulin Secretion. *J. Endocrinol.* **2010**, *205*, 79–86. https://doi.org/10.1677/JOE-09-0311.
8. Tsofack Ngueguim, F.; Christian Esse, E.; Djomeni Dzeufiet, P.D.; Kamkumo Gounoue, R.; Claude Bilanda, D.; Kamtchouing, P.; Dimo, T.T. Oxidised Palm Oil and Sucrose Induced Hyperglycemia in Normal Rats: Effects of *Sclerocarya birrea* Stem Barks Aqueous Extract. *BMC Complement. Altern. Med.* **2016**, *16*, 47. https://doi.org/10.1186/s12906-016-1009-0.
9. Ojewole, J.A.O. Hypoglycemic Effect of *Sclerocarya birrea* [(A. Rich.) Hochst.] [Anacardiaceae] Stem-Bark Aqueous Extract in Rats. *Phytomedicine* **2003**, *10*, 675–681. https://doi.org/10.1078/0944-7113-00295.
10. Sewani-Rusike, C.R.; Ntongazana, O.; Engwa, G.A.; Musarurwa, H.T.; Nkeh-Chungag, B.N. Sclerocarya birrea Fruit Peel Ameliorates Diet-Induced Obesity and Selected Parameters of Metabolic Syndrome in Female Wistar Rats. Pharmacogn. Mag. 2021, 17, 482–491. https://doi.org/10.4103/pm.pm_546_20.
