# Supplementary material for: Systematic Review and Meta-Analysis of Sclerocarya birrea on Metabolic Disorders: Evidence from Preclinical Studies
Source: Metabolites. 2024 Nov 12;14(11):615. doi: 10.3390/metabo14110615 (PMC11596559; doi:10.3390/metabo14110615)
Supplement: Supplementary file 1 [file metabolites-14-00615-s001.zip › Table S2.pdf]

Table S2. Raw Data for Hyperglycemic Control Group

Reference	Species/Strain	Duration	n	Mean before	SD	Mean after 1h	SD	Change after 1h	SD	Mean after 2-4h	SD	Change after 2-4h	SD
Dimo et al. (2007)	Rat/Wistar	0-3h	6	524,00	23,94	507,96	24,84	-16,04	15,45	498,96	30,96	-25,04	18,59
Gondwe et al. (2008)	Rat/Wistar	0-3h	8	459,00	18,00	558,00	21,60	99,00	12,98	513,00	19,80	54,00	12,07
Mogale et al. (2011)	Rat/Wistar	Basal-3h	6	255,60	9,00	374,40	12,60	118,80	7,64	315,00	10,80	59,40	6,49
Ndifossap et al. (2010)	Rat/Wistar	Basal-3h	6	248,00	6,00	394,00	9,00	146,00	5,53	322,00	7,00	74,00	4,22
Sewani-Rusike et al. (2021)	Rat/Wistar	0-2 h	6	109,80	3,60	145,80	5,40	36,00	3,32	109,80	3,60	0,00	2,28
Ojewole et al. (2003)	BALB/c mice	0-4 h	8	560,45	24,55	558,51	24,55	-1,94	15,53	558,50	25,41	-1,95	15,82

References:

1. Dimo, T.; Rakotonirina, S. V.; Tan, P. V.; Azay, J.; Dongo, E.; Kamtchouing, P.; Cros, G. Effect of *Sclerocarya birrea* (Anacardiaceae) Stem Bark Methylene Chloride/Methanol Extract on Streptozotocin-Diabetic Rats. *J. Ethnopharmacol.* **2007**, *110*, 434–438. https://doi.org/10.1016/j.jep.2006.10.020.
2. Gondwe, M.; Kamadyaapa, D.R.; Tufts, M.; Chuturgoon, A.A.; Musabayane, C.T. *Sclerocarya birrea* [(A. Rich.) Hochst.] [Anacardiaceae] Stem-Bark Ethanolic Extract (SBE) Modulates Blood Glucose, Glomerular Filtration Rate (GFR) and Mean Arterial Blood Pressure (MAP) of STZ-Induced Diabetic Rats. *Phytomedicine* **2008**, *15*, 699–709. https://doi.org/10.1016/j.phymed.2008.02.004.
3. Mogale, M.A.; Lebelo, S.L.; Thovhogi, N.; de Freitas, A.N.; Shai, L.J. α-Amylase and α-Glucosidase Inhibitory Effects of *Sclerocarya birrea* [(A. Rich.) Hochst.] Subspecies *Caffra* (Sond) Kokwaro (Anacardiaceae) Stem-Bark Extracts. *Afr. J. Biotechnol.* **2011**, *10*, 15033–15039. https://doi.org/10.5897/AJB11.1408.
4. Makom Ndifossap, I.G.; Frigerio, F.; Casimir, M.; Ngueguim Tsofack, F.; Dongo, E.; Kamtchouing, P.; Dimo, T.T.; Maechler, P.; Ndifossap, I.G.M.; Frigerio, F.; et al. *Sclerocarya birrea* (Anacardiaceae) Stem-Bark Extract Corrects Glycaemia in Diabetic Rats and Acts on Beta-Cells by Enhancing Glucose-Stimulated Insulin Secretion. *J. Endocrinol.* **2010**, *205*, 79–86. https://doi.org/10.1677/JOE-09-0311.
5. Ojewole, J.A.O. Hypoglycemic Effect of *Sclerocarya birrea* [(A. Rich.) Hochst.] [Anacardiaceae] Stem-Bark Aqueous Extract in Rats. *Phytomedicine* **2003**, *10*, 675–681. https://doi.org/10.1078/0944-7113-00295.
