# Supplementary material for: Systematic Review and Meta-Analysis of Sclerocarya birrea on Metabolic Disorders: Evidence from Preclinical Studies
Source: Metabolites. 2024 Nov 12;14(11):615. doi: 10.3390/metabo14110615 (PMC11596559; doi:10.3390/metabo14110615)
Supplement: Supplementary file 1 [file metabolites-14-00615-s001.zip › Table S3.pdf]

Table S3. Raw Data for SB Low Dose

Reference	Species/Strain	Duration	n	Mean before	SD	Mean after 1h	SD	Change after 1h	SD	Mean after 2-4h	SD	Change after 2-4h	SD
Dimo et al. (2007)	Rat/Wistar	0-3h	6	470,9	21,96	443,88	25,92	-27,02	15,60	373,86	25,92	-97,04	15,60
Gondwe et al. (2008)	Rat/Wistar	0-3h	8	511,2	19,8	356,4	14,4	-154,80	11,97	333	12,6	-178,20	12,31
Ndifossap et al. (2010)	Rat/Wistar	Basal-3h	6	248	6	318	7	70,00	4,22	192	6	-56,00	3,79
Sewani-Rusike et al. (2021)	Rat/Wistar	0-2 h	6	5,9	3,6	135	5,4	28,80	3,32	106,2	3,6	0,00	2,28

1. Dimo, T.; Rakotonirina, S. V.; Tan, P. V.; Azay, J.; Dongo, E.; Kamtchouing, P.; Cros, G. Effect of *Sclerocarya birrea* (Anacardiaceae) Stem Bark Methylene Chloride/Methanol Extract on Streptozotocin-Diabetic Rats. *J. Ethnopharmacol.* **2007**, *110*, 434–438. https://doi.org/10.1016/j.jep.2006.10.020.
2. Gondwe, M.; Kamadyaapa, D.R.; Tufts, M.; Chuturgoon, A.A.; Musabayane, C.T. *Sclerocarya birrea* [(A. Rich.) Hochst.] [Anacardiaceae] Stem-Bark Ethanolic Extract (SBE) Modulates Blood Glucose, Glomerular Filtration Rate (GFR) and Mean Arterial Blood Pressure (MAP) of STZ-Induced Diabetic Rats. *Phytomedicine* **2008**, *15*, 699–709. https://doi.org/10.1016/j.phymed.2008.02.004.
3. Makom Ndifossap, I.G.; Frigerio, F.; Casimir, M.; Ngueguim Tsofack, F.; Dongo, E.; Kamtchouing, P.; Dimo, T.T.; Maechler, P.; Ndifossap, I.G.M.; Frigerio, F.; et al. *Sclerocarya birrea* (Anacardiaceae) Stem-Bark Extract Corrects Glycaemia in Diabetic Rats and Acts on Beta-Cells by Enhancing Glucose-Stimulated Insulin Secretion. *J. Endocrinol.* **2010**, *205*, 79–86. https://doi.org/10.1677/JOE-09-0311.
4. Sewani-Rusike, C.R.; Ntongazana, O.; Engwa, G.A.; Musarurwa, H.T.; Nkeh-Chungag, B.N. Sclerocarya birrea Fruit Peel Ameliorates Diet-Induced Obesity and Selected Parameters of Metabolic Syndrome in Female Wistar Rats. Pharmacogn. Mag. 2021, 17, 482–491. https://doi.org/10.4103/pm.pm_546_20.
