# Supplementary material for: Systematic Review and Meta-Analysis of Sclerocarya birrea on Metabolic Disorders: Evidence from Preclinical Studies
Source: Metabolites. 2024 Nov 12;14(11):615. doi: 10.3390/metabo14110615 (PMC11596559; doi:10.3390/metabo14110615)
Supplement: Supplementary file 1 [file metabolites-14-00615-s001.zip › Table S4.pdf]

Table S4. Raw Data for SB High Dose

Reference	Species/Strain	Duration	n	Mean before	SD	Mean after 1h	SD	Change_1h	SD	Mean after 2-4h	SD	Change_2h-4h	SD
Dimo et al. (2007)	Rat/Wistar	0-3h	6	263,00	16,92	232,92	13,86	-30,08	10,16	205,92	18,00	-57,08	11,09
Gondwe et al. (2008)	Rat/Wistar	0-3h	8	516,60	19,80	295,20	12,60	-221,40	12,31	311,40	10,80	-205,20	12,90
Mogale et al. (2011)	Rat/Wistar	Basal-3h	6	261,00	9,00	329,40	10,80	68,40	6,49	291,60	9,00	30,60	5,69
Ndifossap et al. (2010)	Rat/Wistar	Basal-3h	6	248,00	6,00	276,00	8,00	28,00	4,82	142,00	5,00	-106,00	3,61
Sewani-Rusike et al. (2021)	Rat/Wistar	0-2 h	6	93,60	3,60	127,80	5,40	34,20	3,32	118,80	3,60	25,20	2,28
Ojewole et al. (2003)	BALB/c mice	0-4 h	8	550,51	25,41	464,37	23,28	-86,14	15,53	281,56	20,45	-268,95	15,25

1. Dimo, T.; Rakotonirina, S. V.; Tan, P. V.; Azay, J.; Dongo, E.; Kamtchouing, P.; Cros, G. Effect of *Sclerocarya birrea* (Anacardiaceae) Stem Bark Methylene Chloride/Methanol Extract on Streptozotocin-Diabetic Rats. *J. Ethnopharmacol.* **2007**, *110*, 434–438. https://doi.org/10.1016/j.jep.2006.10.020.
2. Gondwe, M.; Kamadyaapa, D.R.; Tufts, M.; Chuturgoon, A.A.; Musabayane, C.T. *Sclerocarya birrea* [(A. Rich.) Hochst.] [Anacardiaceae] Stem-Bark Ethanolic Extract (SBE) Modulates Blood Glucose, Glomerular Filtration Rate (GFR) and Mean Arterial Blood Pressure (MAP) of STZ-Induced Diabetic Rats. *Phytomedicine* **2008**, *15*, 699–709. https://doi.org/10.1016/j.phymed.2008.02.004.
3. Mogale, M.A.; Lebelo, S.L.; Thovhogi, N.; de Freitas, A.N.; Shai, L.J. α-Amylase and α-Glucosidase Inhibitory Effects of *Sclerocarya birrea* [(A. Rich.) Hochst.] Subspecies *Caffra* (Sond) Kokwaro (Anacardiaceae) Stem-Bark Extracts. *Afr. J. Biotechnol.* **2011**, *10*, 15033–15039. https://doi.org/10.5897/AJB11.1408.
4. Makom Ndifossap, I.G.; Frigerio, F.; Casimir, M.; Ngueguim Tsofack, F.; Dongo, E.; Kamtchouing, P.; Dimo, T.T.; Maechler, P.; Ndifossap, I.G.M.; Frigerio, F.; et al. *Sclerocarya birrea* (Anacardiaceae) Stem-Bark Extract Corrects Glycaemia in Diabetic Rats and Acts on Beta-Cells by Enhancing Glucose-Stimulated Insulin Secretion. *J. Endocrinol.* **2010**, *205*, 79–86. https://doi.org/10.1677/JOE-09-0311.
5. Sewani-Rusike, C.R.; Ntongazana, O.; Engwa, G.A.; Musarurwa, H.T.; Nkeh-Chungag, B.N. Sclerocarya birrea Fruit Peel Ameliorates Diet-Induced Obesity and Selected Parameters of Metabolic Syndrome in Female Wistar Rats. Pharmacogn. Mag. 2021, 17, 482–491. https://doi.org/10.4103/pm.pm_546_20.
6. Ojewole, J.A.O. Hypoglycemic Effect of *Sclerocarya birrea* [(A. Rich.) Hochst.] [Anacardiaceae] Stem-Bark Aqueous Extract in Rats. *Phytomedicine* **2003**, *10*, 675–681. https://doi.org/10.1078/0944-7113-00295.
