# Supplementary material for: Systematic Review and Meta-Analysis of Sclerocarya birrea on Metabolic Disorders: Evidence from Preclinical Studies
Source: Metabolites. 2024 Nov 12;14(11):615. doi: 10.3390/metabo14110615 (PMC11596559; doi:10.3390/metabo14110615)
Supplement: Supplementary file 1 [file metabolites-14-00615-s001.zip › Table S5.pdf]

Table S5. Raw Data for Chronic Consumption of SB

Reference	Species/Strain	Duration	n	Control group						Low dose group					
				Mean before	SD	Mean after	SD	Change after	SD	Mean before	SD	Mean after	SD	Change after	SD
Dimo et al. (2007)	Rat/Wistar	3 weeks	6	523,98	23,94	671,04	29,88	147,06	17,93	470,88	21,96	694,98	32,94	224,10	20,25
Gondwe et al. (2008)	Rat/Wistar	5 weeks	8	459,00	18,00	451,80	23,40	-7,20	14,06	511,20	19,80	325,80	18,00	-185,40	12,07
Ndifossap et al. (2010)	Rat/Wistar	2 weeks	6	246,00	6,00	135,00	4,00	-78,00	3,61	246,00	6,00	135,00	4,00	-111,00	3,69

1. Dimo, T.; Rakotonirina, S. V.; Tan, P. V.; Azay, J.; Dongo, E.; Kamtchouing, P.; Cros, G. Effect of *Sclerocarya birrea* (Anacardiaceae) Stem Bark Methylene Chloride/Methanol Extract on Streptozotocin-Diabetic Rats. *J. Ethnopharmacol.* **2007**, *110*, 434–438. https://doi.org/10.1016/j.jep.2006.10.020.
2. Gondwe, M.; Kamadyaapa, D.R.; Tufts, M.; Chuturgoon, A.A.; Musabayane, C.T. *Sclerocarya birrea* [(A. Rich.) Hochst.] [Anacardiaceae] Stem-Bark Ethanolic Extract (SBE) Modulates Blood Glucose, Glomerular Filtration Rate (GFR) and Mean Arterial Blood Pressure (MAP) of STZ-Induced Diabetic Rats. *Phytomedicine* **2008**, *15*, 699–709. https://doi.org/10.1016/j.phymed.2008.02.004.
3. Makom Ndifossap, I.G.; Frigerio, F.; Casimir, M.; Ngueguim Tsofack, F.; Dongo, E.; Kamtchouing, P.; Dimo, T.T.; Maechler, P.; Ndifossap, I.G.M.; Frigerio, F.; et al. *Sclerocarya birrea* (Anacardiaceae) Stem-Bark Extract Corrects Glycaemia in Diabetic Rats and Acts on Beta-Cells by Enhancing Glucose-Stimulated Insulin Secretion. *J. Endocrinol.* **2010**, *205*, 79–86. https://doi.org/10.1677/JOE-09-0311.
