# Supplementary figures and images for: Systematic Review and Meta-Analysis of Sclerocarya birrea on Metabolic Disorders: Evidence from Preclinical Studies
Source: Metabolites. 2024 Nov 12;14(11):615. doi: 10.3390/metabo14110615 (PMC11596559; doi:10.3390/metabo14110615)

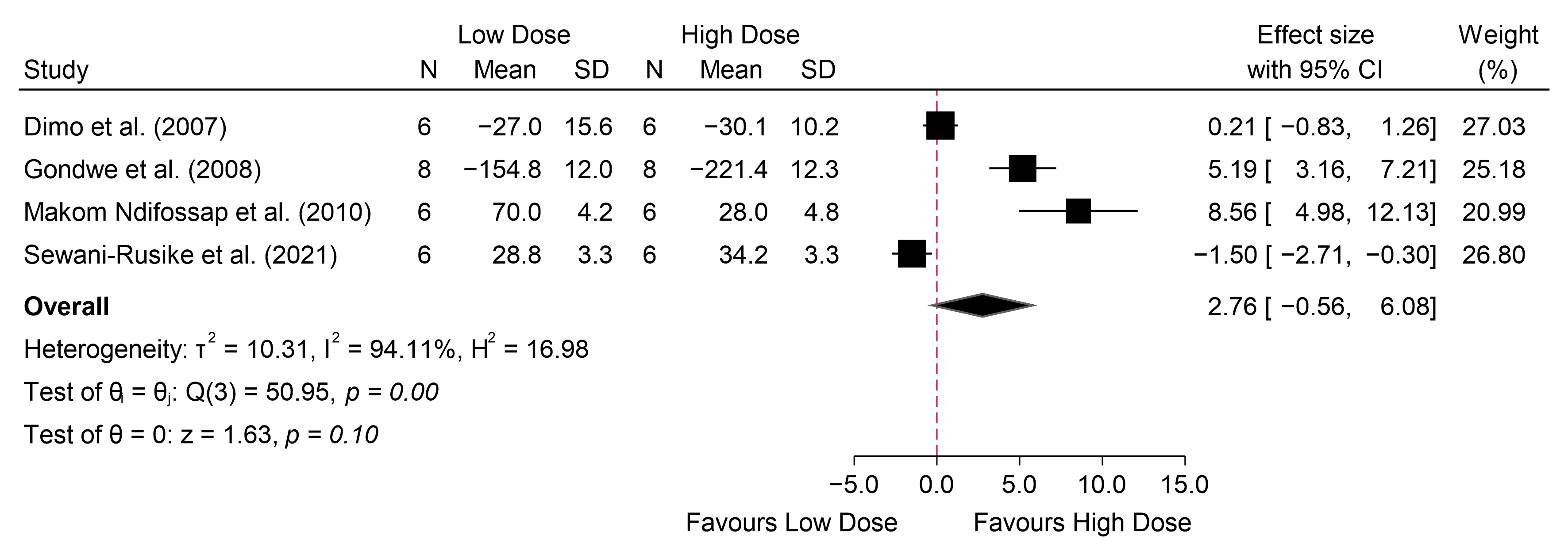

Supplement: Supplementary file 1 [file metabolites-14-00615-s001.zip › Figure S1.tif]

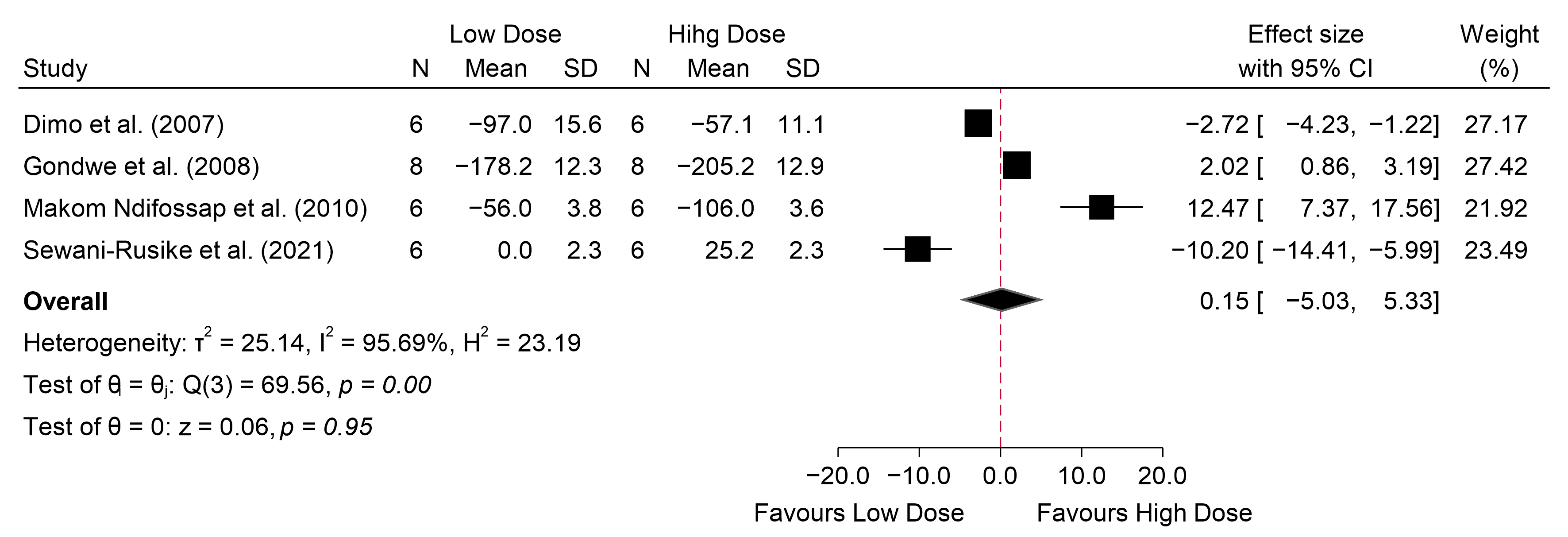

Supplement: Supplementary file 1 [file metabolites-14-00615-s001.zip › Figure S2.tif]

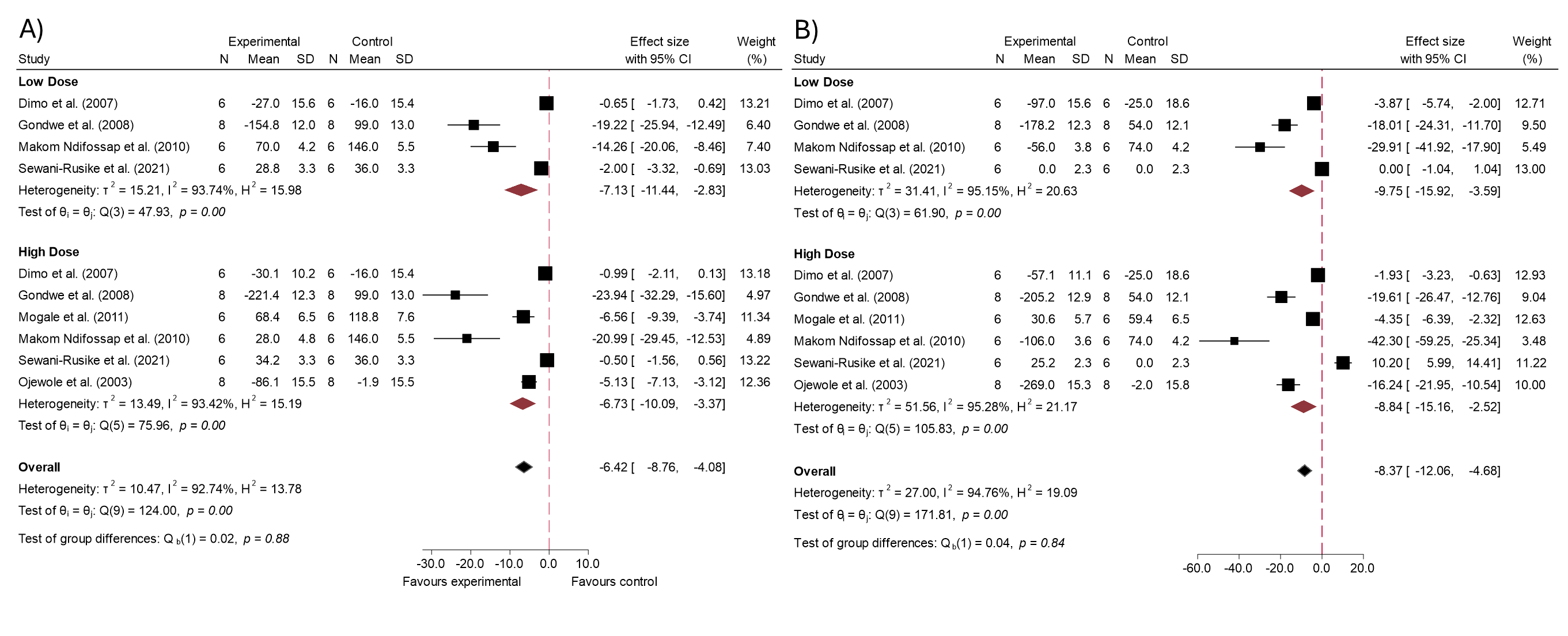

Supplement: Supplementary file 1 [file metabolites-14-00615-s001.zip › Figure S3.tif]

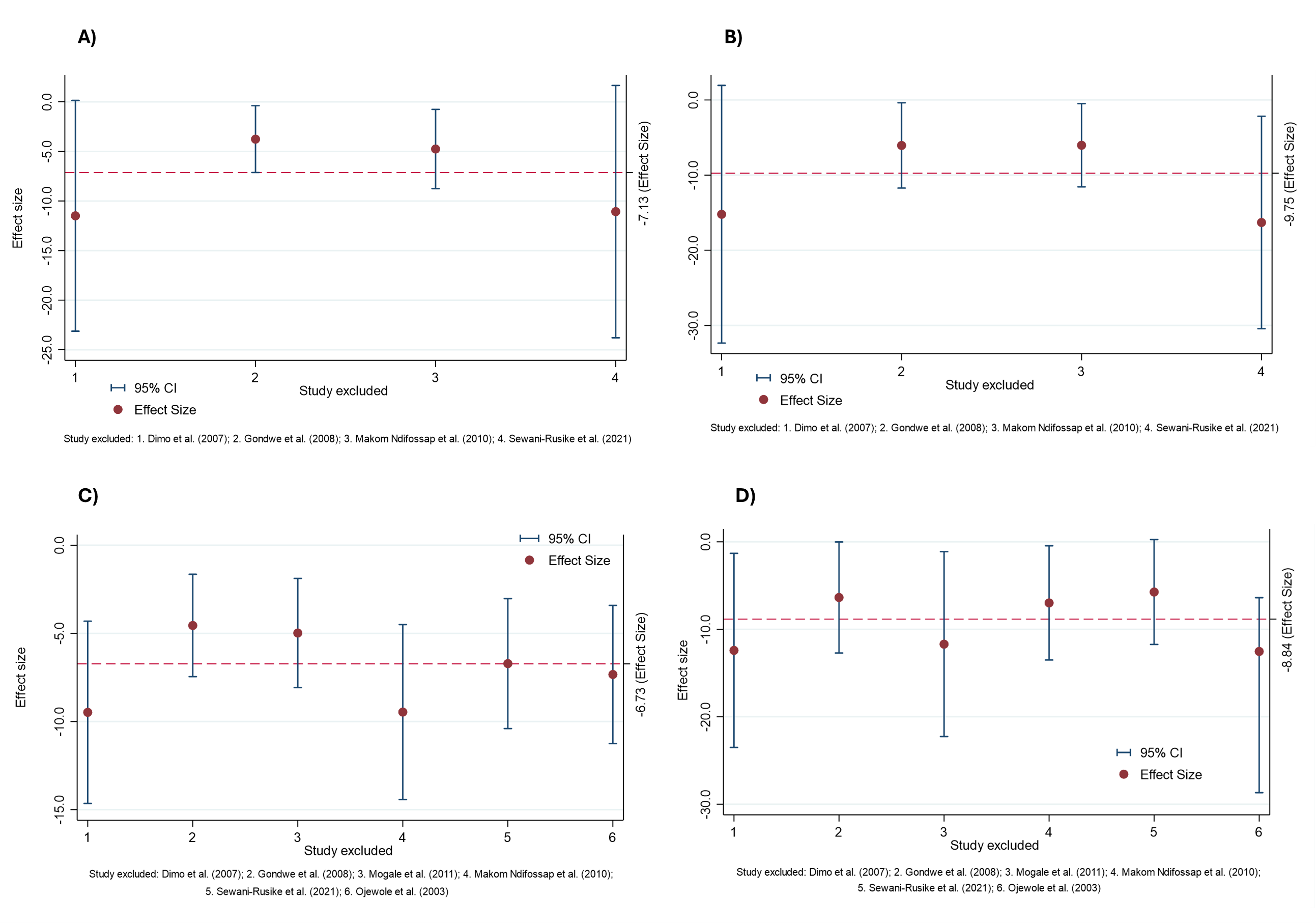

Supplement: Supplementary file 1 [file metabolites-14-00615-s001.zip › Figure S4.tif]

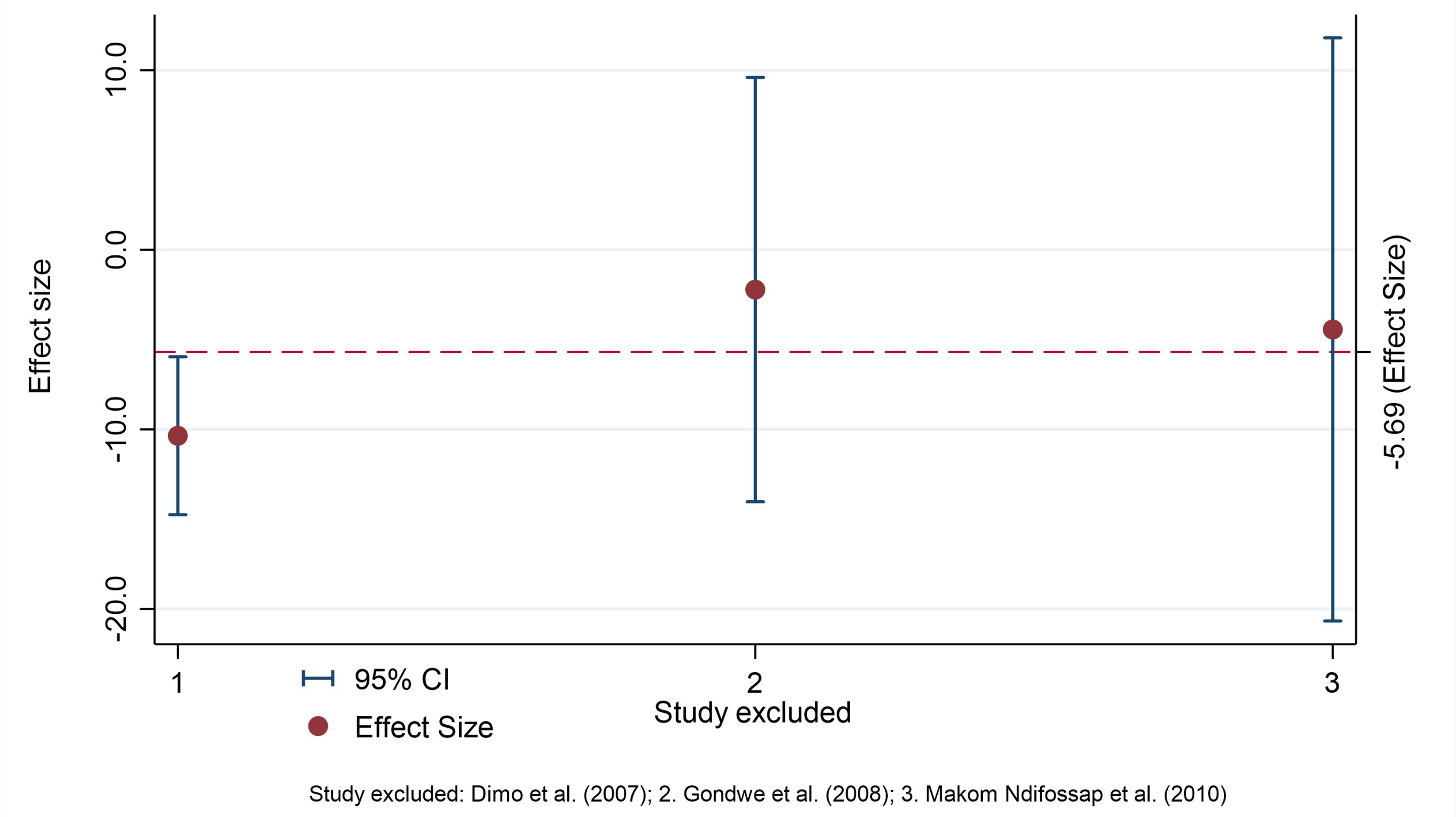

Supplement: Supplementary file 1 [file metabolites-14-00615-s001.zip › Figure S5.tif]

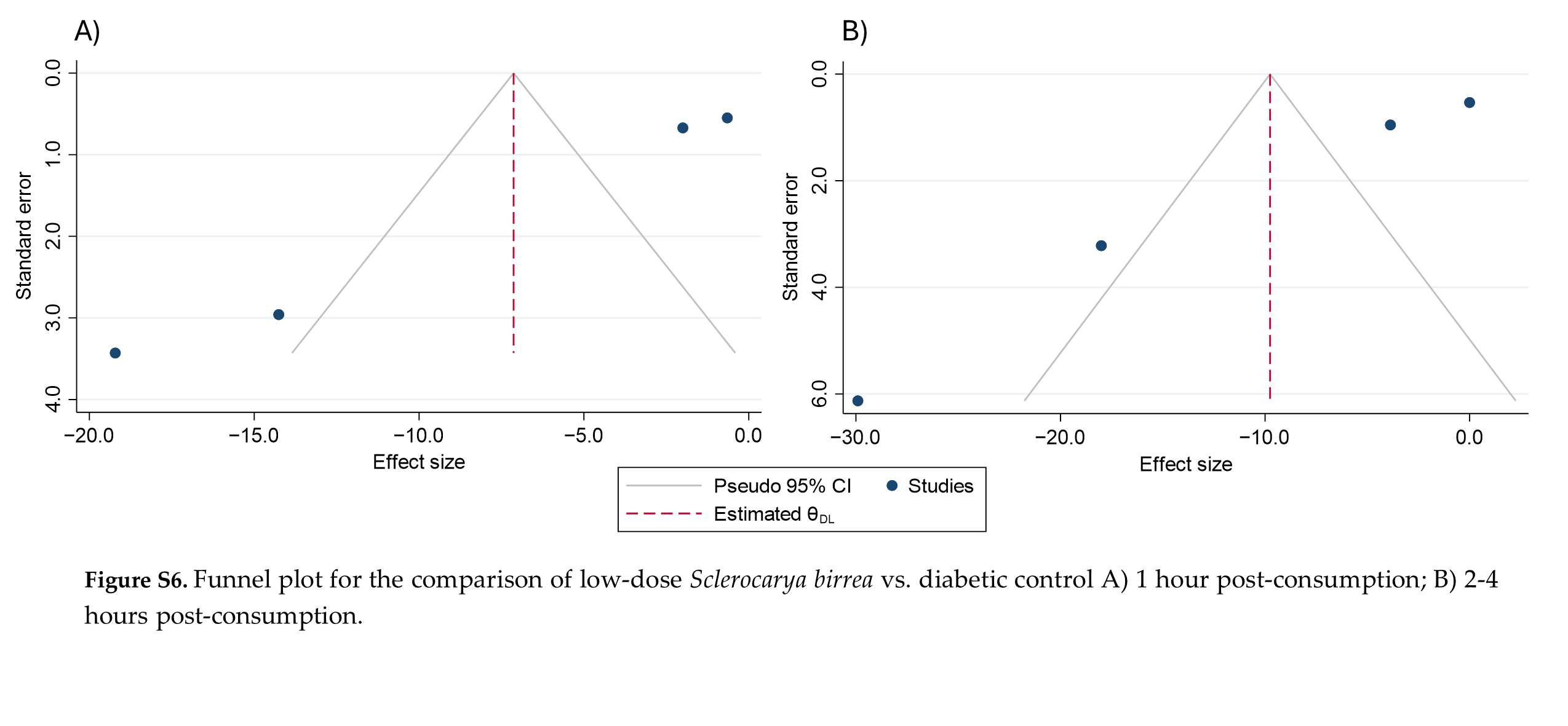

Supplement: Supplementary file 1 [file metabolites-14-00615-s001.zip › Figure S6.tif]

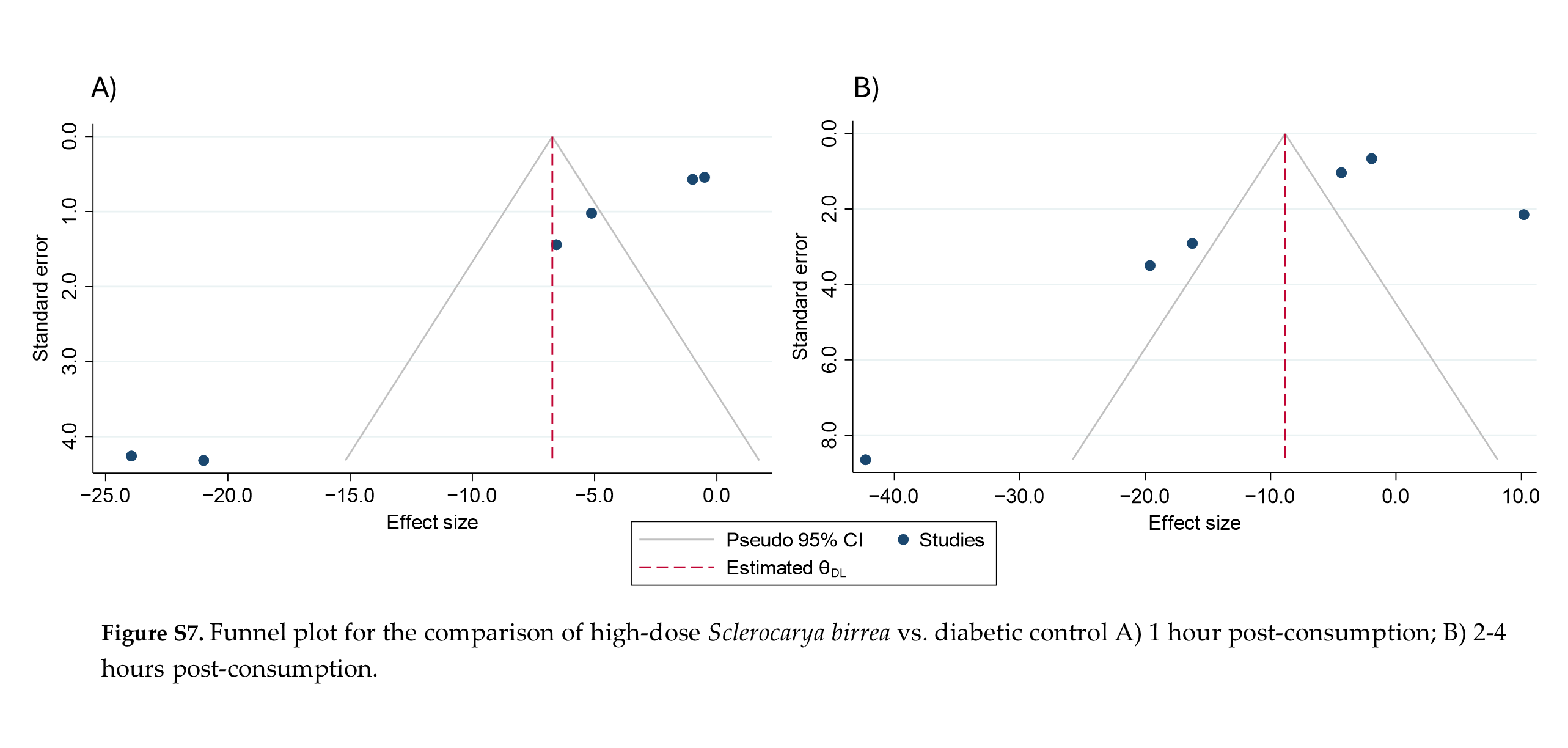

Supplement: Supplementary file 1 [file metabolites-14-00615-s001.zip › Figure S7.tif]

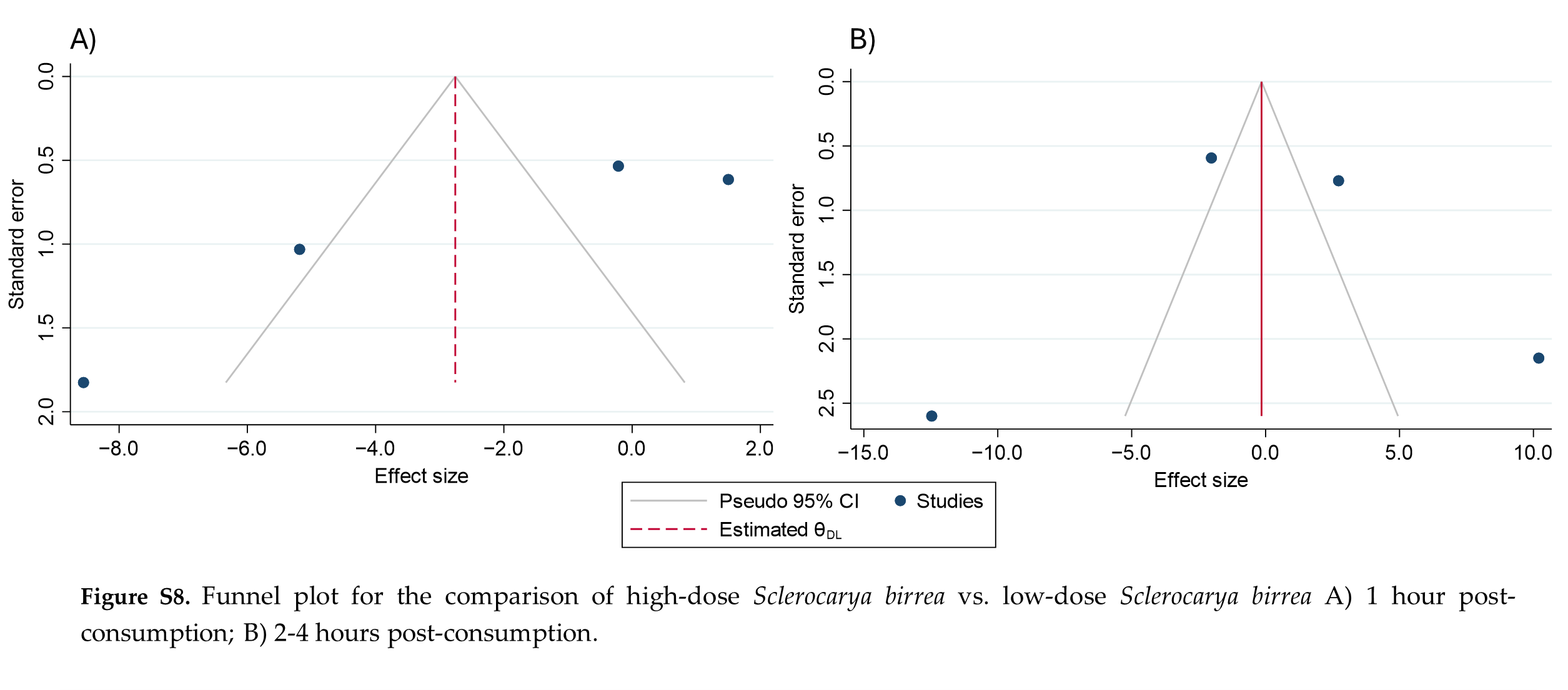

Supplement: Supplementary file 1 [file metabolites-14-00615-s001.zip › Figure S8.tif]

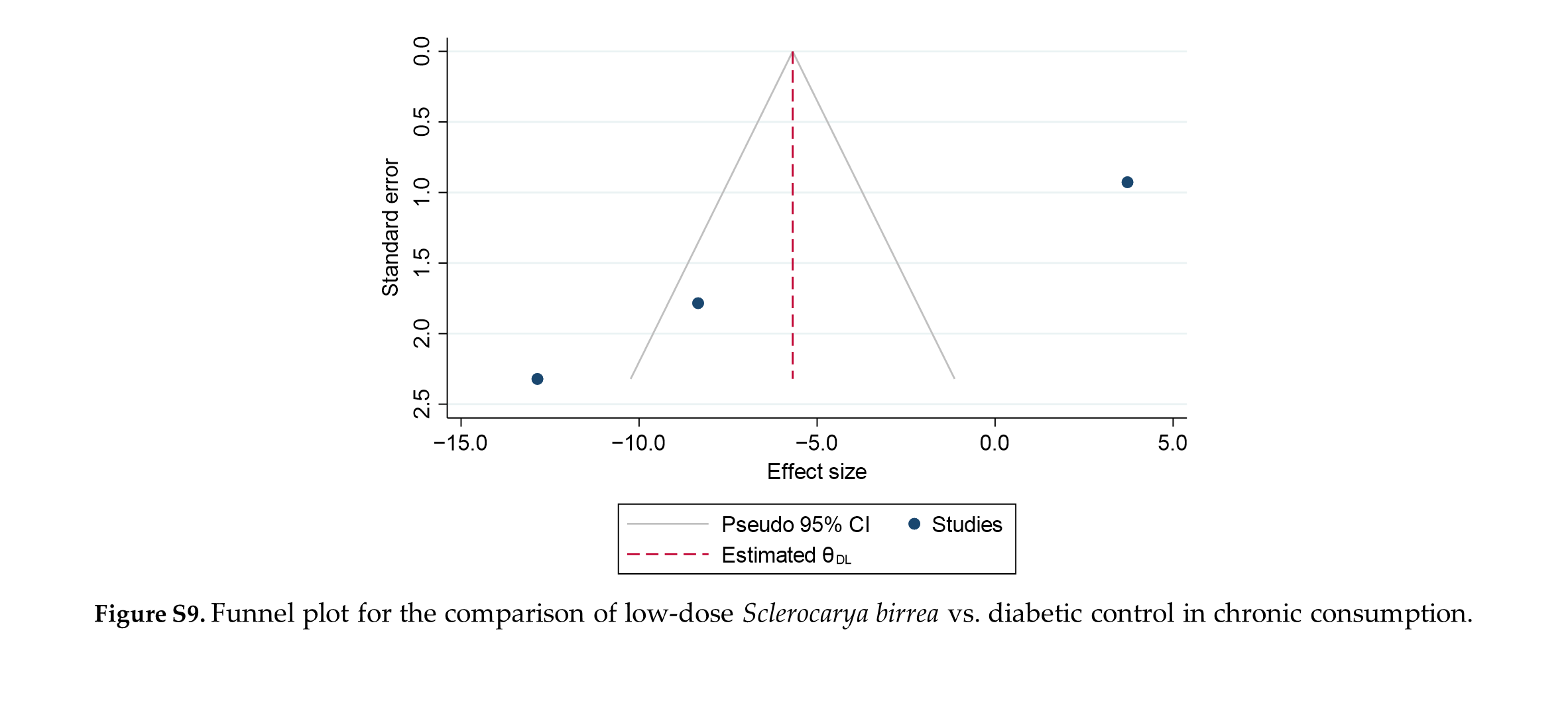

Supplement: Supplementary file 1 [file metabolites-14-00615-s001.zip › Figure S9.tif]
